# Neutrophils use superoxide to control bacterial infection at a distance

**DOI:** 10.1371/journal.ppat.1007157

**Published:** 2018-07-17

**Authors:** Quang Tien Phan, Tamara Sipka, Catherine Gonzalez, Jean-Pierre Levraud, Georges Lutfalla, Mai Nguyen-Chi

**Affiliations:** 1 DIMNP, CNRS, Univ Montpellier, Montpellier, France; 2 Dept of Biological Sciences National University of Singapore, Singapore; 3 Macrophages et Développement de l’Immunité, Institut Pasteur, Paris, France; 4 CNRS, UMR3738, Paris, France; University of California Davis School of Medicine, UNITED STATES

## Abstract

Understanding the roles of neutrophils and macrophages in fighting bacterial infections is a critical issue in human pathologies. Although phagocytic killing has been extensively studied, little is known about how bacteria are eliminated extracellularly in live vertebrates. We have recently developed an infection model in the zebrafish embryo in which leukocytes cannot reach the injected bacteria. When *Escherichia coli* bacteria are injected within the notochord, both neutrophils and macrophages are massively recruited during several days, but do not infiltrate the infected tissue presumably because of its tough collagen sheath. Nevertheless, the bacteria are killed during the first 24 hours, and we report here that neutrophils, but not macrophages are involved in the control of the infection. Using genetic and chemical approaches, we show that even in absence of phagocytosis, the bactericidal action relies on NADPH oxidase-dependent production of superoxide in neutrophils. We thus reveal a host effector mechanism mediated by neutrophils that eliminates bacteria that cannot be reached by phagocytes and that is independent of macrophages, NO synthase or myeloperoxidase.

## Author summary

Deciphering the defence mechanisms of leukocytes remains a challenge for public health. Although phagocytic killing has been extensively studied, little is known about how bacteria are eliminated extracellularly in live vertebrates. Herein we use the notochord infection model in the zebrafish embryo to describe how leukocytes eliminate distant bacteria that are inaccessible for phagocytosis. In this context neutrophils but not macrophages are instrumental for bacterial clearance and larva survival. We then found that neutrophil bactericidal action relies on the NADPH oxidase dependent production of superoxide and is independent of NO synthase or myeloperoxidase.

## Introduction

The innate immune system is the first line of defence of the host. It includes large phagocytes (such as macrophages and granulocytes) equipped with a battery of weapons to destroy the invader within minutes or hours. Since the seminal work of Elie Metchnikoff [1], the defence mechanisms relying on leukocytes remain a challenging subject. When microbes penetrate the epithelial barrier, macrophages and neutrophils are rapidly recruited and upon contact, engulf the bacteria into a vacuole called a phagosome that fuses with intracellular granules or lysosomes to form a lytic vacuole in which bacteria may be killed by a wide variety of mechanisms involving chemicals and enzymes [2,3]. Non-oxidative effectors include antimicrobial proteins, while the oxygen-dependent mechanism, also known as the respiratory burst, involves the generation of reactive oxygen species (ROS) [4,5,6]. ROS production inside the phagocytic vacuole involves NADPH oxidase and the major ROS, superoxide (O_2_^-^) and hydrogen peroxide (H_2_O_2_), can directly or indirectly promote the death of the microbe, according to the nature of the pathogens [7,8]. Nitric oxide (NO), produced by NO synthase, can contribute to microbicidal activity and is essential for the defence against intracellular organisms such as *Salmonella enterica* and mycobacteria [9,10].

Many microbes manage to survive within macrophages after phagocytosis. While some cope with the phagolysosomal conditions (*S*. *enterica serovar Typhimurium* [11]), others like *Listeria*, *Shigella* and some mycobacteria [12,13,14] are able to block the maturation of the phagosome or even to escape from these compartments. Host cells, however, have developed counter strategies to fight cytosolic bacteria including directing them to autophagosomes [15].

While microbe killing inside the phagosome has been extensively studied, it is less well understood how phagocytes are capable of killing microbes extracellularly in whole organisms. Neutrophils can fight bacterial pathogens without phagocytosis either by release of toxic granule contents (degranulation) [16] or by expelling neutrophil extracellular traps (NETs), which are networks of extracellular fibres built upon expulsion of chromatin [17]. However, events such as these are very hard to disentangle from phagocytosis-mediated killing in the full context of tissue infection.

Thanks to its transparency and genetic amenability, the zebrafish embryo is a useful model for the study of host/pathogen interactions *in vivo*. The zebrafish model has been used to evaluate the respective roles of neutrophils and macrophages in eliminating invading bacteria [10,18,19]; this relies not only on the nature of the invading microbe, but also on the route and anatomical site of infection. One striking observation was that macrophages are very efficient at engulfing microbes from body fluids (“flypaper” strategy) while neutrophils may be very efficient at clearing surface associated microbes in a “vacuum-cleaner”-like behaviour [20].

We have recently developed an infection model in the zebrafish embryo in which the bacteria are trapped in a tissue in which macrophages and neutrophils cannot enter. When non-pathogenic *Escherichia coli* (*E*. *coli*) bacteria are injected in the notochord, the swollen rod that provides axial stiffness to the developing embryo, they slide between notochord cells and the thick cylindrical collagen sheath that encases the cord. Although unable to thread their way through this envelope, neutrophils and macrophages are massively recruited all along the infected notochord where they stay in a highly activated state for days. Interestingly, these inaccessible bacteria are cleared within the first 24 hours [21].

Here we address the mechanisms of *E*. *coli* clearance in the notochord infection model where professional phagocytes cannot directly encounter the injected bacteria. We first investigate whether macrophages or neutrophils are involved in this clearance and then investigate the nature of the molecules instrumental for bacterial killing.

## Results

### Macrophages are not required for the control of *E*. *coli* infection in the notochord

**Fig 1:**
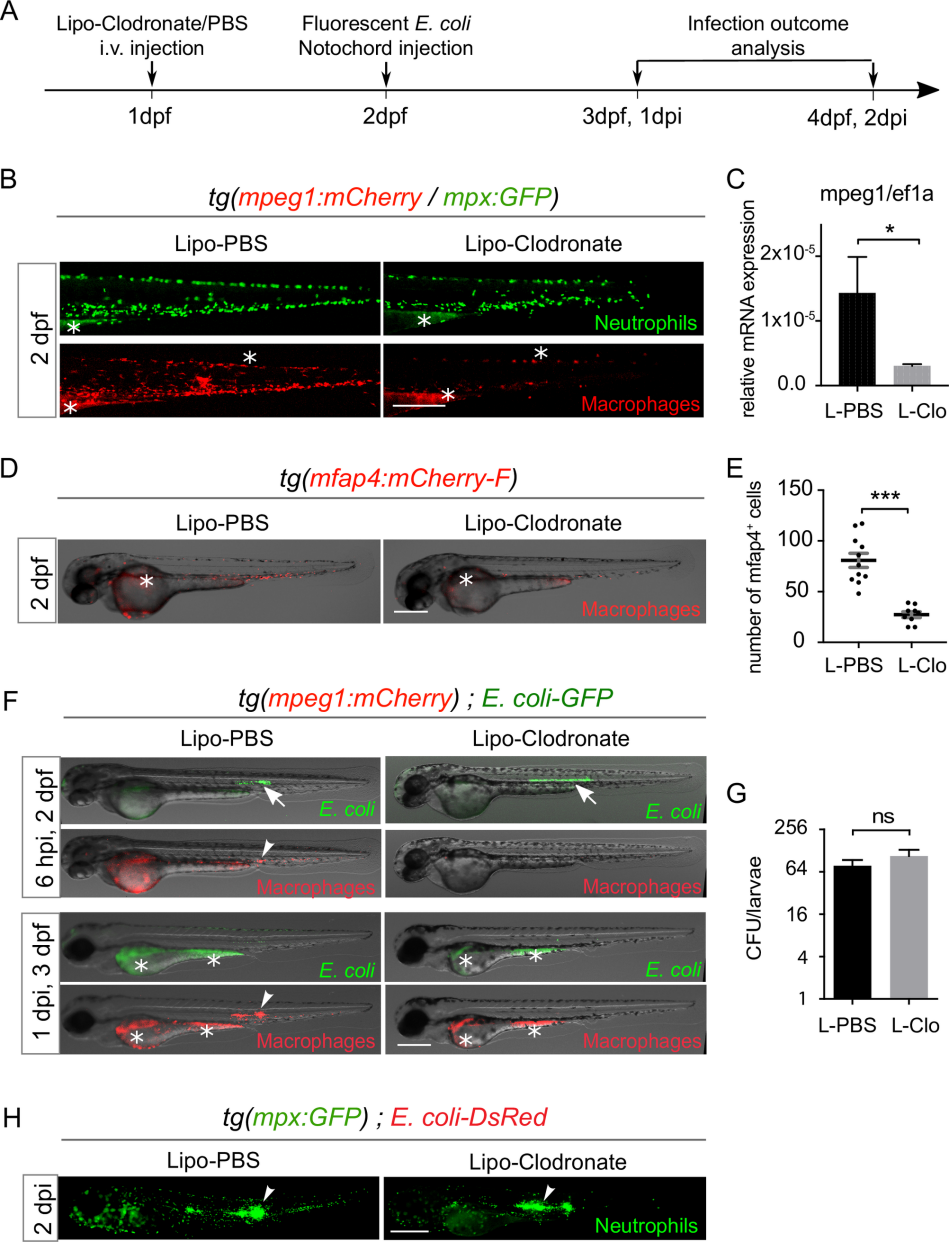
Macrophages are not involved in the clearance of bacteria injected within the notochord. (A) Experimental scheme. One dpf *tg(mpeg1*:*mCherry-F/mpx*:*GFP) or tg(mfap4*:*mCherry-F) or tg(mpeg1*:*mCherry-F)* embryos were *i*.*v*. injected with Lipo-Clodronate (L-clo) or Lipo-PBS (L-PBS). Correctly depleted larvae were selected based on the loss of red fluorescent macrophages, and GFP or DsRed expressing *E*. *coli* were injected within their notochord at 2 dpf. The infection outcome was analyzed at 1 and 2 dpi using fluorescence microscopy. (B) Lipo-Clodronate efficiently depletes macrophages without affecting neutrophil population. Experiments were performed as described in (A) on *tg(mpeg1*:*mCherry-F/mpx*:*GFP)*. GFP (neutrophils) and mCherry (macrophages) were analysed by fluorescence microscopy at 2 dpf. (C) qRT-PCR measurement of *mpeg1* mRNA relative to *ef1a* in Lipo-PBS and Lipo-clodronate conditions in whole larvae at 3 dpf (pool of 10 larvae, mean values ± Standard Error of the Mean (SEM), three experiments, Mann Whitney test, one tailed, *P<0.*05*). (D) *Tg(mfap4*:*mCherry-F)* were treated with Lipo-Clodronate or Lipo-PBS as described in (A). mCherry (macrophages) was analysed by fluorescence microscopy at 2 dpf. Representative fluorescence overlaid with brightfield images show macrophage depletion in Lipo-Clodronate treated larvae. (E) Macrophage counts (mfap4^+^ cells) at 2 dpf in indicated conditions (horizontal lines indicate the mean ± SEM, Student test, one-tailed, ***p<0.*001)*. (F) *E*. *coli*-GFP infections in the notochord of *tg(mpeg1*:*mCherry-F)* embryos are cleared in macrophage-depleted embryos. GFP (*E*. *coli*) and mCherry (macrophages) were imaged repeatedly in individual larvae using fluorescence microscopy at 6 hpi and 1 dpi. In both Lipo-PBS and Lipo-clodronate conditions, *E*. *coli*-GFP are present in the notochord at 6 hpi (white arrows) but are cleared at 1 dpi (*N*_*L-PBS*_ *= 5* and *N*_*L-clo*_ *= 9)*. Arrowhead shows the recruitment of macrophage in Lipo-PBS injected larvae. Asterisks show the auto-fluorescence of the yolk. (G) CFU counts at 1 dpi in notochord infected of Lipo-PBS and Lipo-Clodronate treated larvae (mean number of CFU per larva ± SEM, *N*_*L-PBS*_ *= 9* and *N*_*L-clo*_ *= 5*, Mann Whitney test, two tailed, *p>0*.*05*, ns = not significant). (H) *E*. *coli* infections in the notochord of *tg(mpx*:*GFP)* embryos after macrophage depletion with Lipo-Clodronate. GFP (Neutrophils) was imaged in larvae using fluorescence microscopy at 2 dpi (*N*_*L-PBS*_ *= 25* and *N*_*L-clo*_ *= 24)*. Scale bars: 400 μm.

We previously showed that K12 *Escherichia coli* cells injected in the notochord of zebrafish embryos cannot be reached by phagocytes, yet are killed in one day [21]. We confirmed the physical separation of freshly injected K12 from phagocytes by the notochord collagen matrix (S1A and S1B Fig). To verify that this is not a quirk of this laboratory strain, we first compared enteric adherent invasive *E*. *coli* strains, *E*. *coli* AIEC LF82 and its mutant, LF82-ΔlpfA, *E*. *coli* JM83-ΔmsbB strain and laboratory K12 strain in our notochord infection model. We observed that they behaved similarly (S1C and S1D Fig). We therefore went on using the laboratory K12 strain. To investigate the role of macrophages in the observed bacterial clearance, we injected liposome-encapsulated clodronate (Lipo-clodronate) that kills phagocytic macrophages [22,23]. At 1 day post-fertilization (dpf), macrophage/neutrophil dual reporter embryos, *tg(mpeg1*:*mCherry-F)/tg(mpx*:*GFP)*, or macrophage reporter embryos, *tg(mpeg1*:*mCherry-F)*, were injected with 10 nl of Lipo-Clodronate in the posterior caudal vein (intravenous, i.v.). As previously described [22] 24 h after Lipo-Clodronate injection, macrophages were efficiently eliminated without affecting the neutrophil population, nor inducing unspecific toxicity (Fig 1A and 1B). This was correlated with the decrease of *mpeg1* mRNA expression in Lipo-Clodronate treated larvae compared to Lipo-PBS controls, as shown by RT-qPCR (Fig 1C). To further confirm the efficiency of lipo-clodronate to suppress macrophage population, we generated another macrophage reporter line with microfibrillar-associated protein 4 (mfap4) promoter whose expression is strong and stable in zebrafish macrophages [24], i.e. the *tg(mfap4*:*mCherry-F)* line. Injection of Lipo-clodronate in *tg(mfap4*:*mCherry-F)* induced a dramatic reduction in the number of mfap4^+^ cells (Fig 1D and 1E), showing the suitability of this approach to deplete macrophages. Macrophage depleted larvae were selected and injected in the notochord with fluorescent *E*. *coli*. We observed that bacteria were cleared within the first 24 hours post infection (hpi) in both, macrophage-depleted larvae, as well as in control Lipo-PBS injected larvae, as revealed by fluorescence microscopy and CFU counts (Fig 1F and 1G). Importantly, upon notochord infection, neutrophils were normally recruited around the infected notochord regardless of the presence or absence of macrophages (Fig 1H).

To confirm, that macrophages are not fundamental for bacterial clearance in notochord infection model, we ablate macrophages using *tg(mpeg1*:*Gal4 / UAS*:*nfsB-mCherry)* embryos in which *macrophage express gene 1* promoter indirectly drives the expression of *E*. *coli* nitroreductase enzyme in macrophages. Treatment of *tg(mpeg1*:*Gal4/UAS*:*nfsB-mCherry)* embryos with the pro-drug metronidazole (MTZ) at 30 hpf (hours post-fertlilization) specifically decreased macrophage number at 1 and 2 days post-treatment (dpT) (S2A and S2B Fig). *Tg(mpeg1*:*Gal4/UAS*:*nfsB-mCherry)* were then infected with *E*. *coli-GFP* at 2 dpf in the notochord. MTZ-mediated macrophage depletion did not impact the bacterial burden at 1 dpi (day post-infection) as shown by Fluorescent Pixel Counts (FPC) (S2C and S2D Fig). Altogether, these data show that macrophages are not required for bacterial clearance in this model.

### Neutrophils are essential for the control of notochord infection by *E*. *coli*

**Fig 2:**
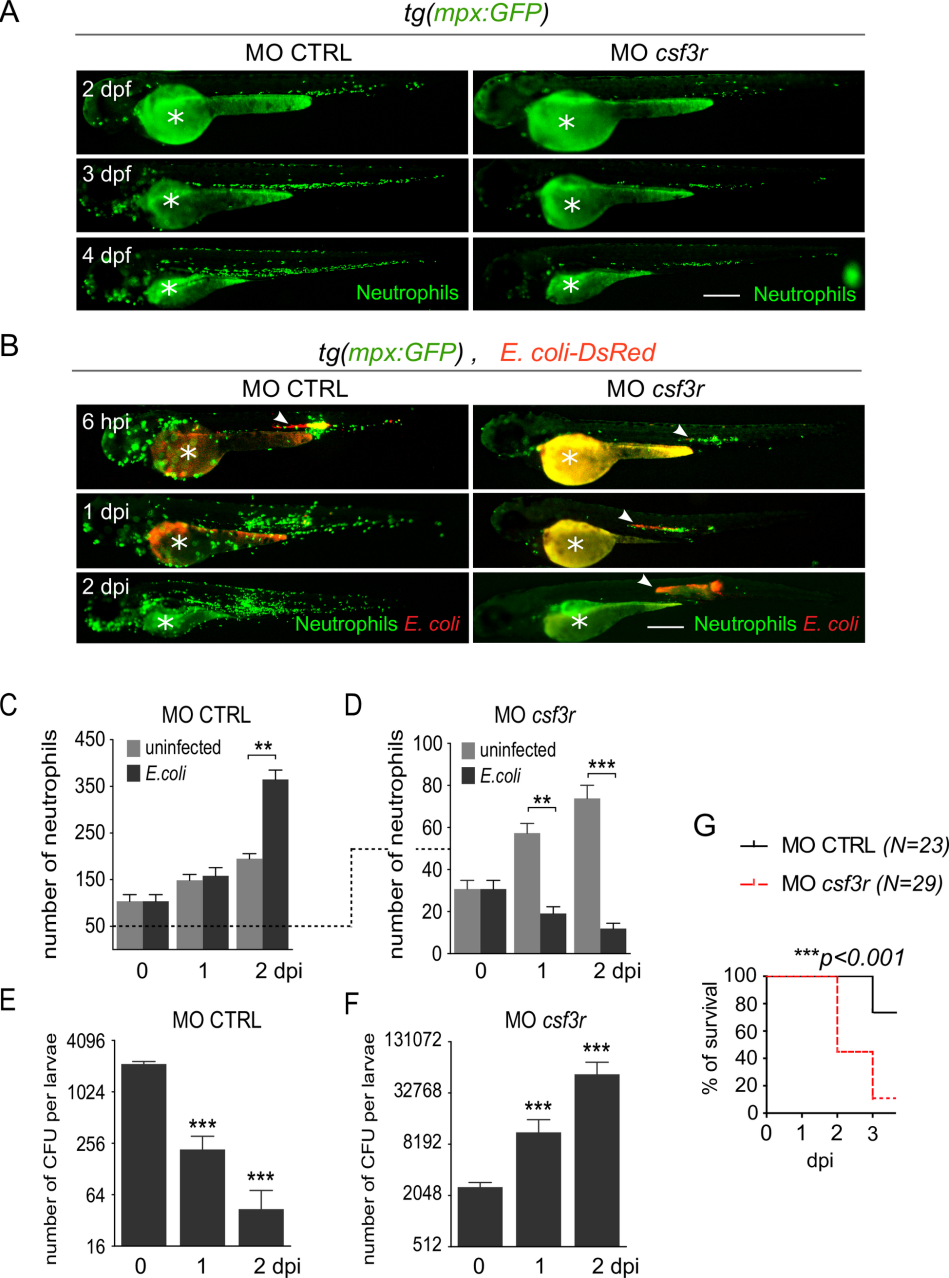
Neutrophils are essential for bacterial clearance. *Tg(mpx*:*GFP)* embryos were injected at the one cell stage with either *csf3r* morpholino (MO *csf3r*) to induce neutrophil depletion or a control morpholino (MO CTRL). (A) Steady-state neutrophil populations were imaged repeatedly in individual morphants using GFP fluorescence in both MO *csf3r* and control conditions between 2 and 4 dpf. (B) Fluorescent *E*. *coli*-DsRed were injected in the notochord of *csf3r* and CTRL morphants. GFP (Neutrophils) and DsRed (*E*. *coli*) fluorescence were imaged at indicated time points. *E*. *coli*-DsRed (red) disappeared from 1 dpi in control embryos (left panels), while it increased in *csf3r* morphants at 1 and 2 dpi (white arrowheads) with a concomitant decrease in neutrophil number (green). Scale bars: 400 μm. (C, D) Quantification of total neutrophils in CTRL (C) and *csf3r* (D) morphants at the indicated time points following PBS (light grey columns) or *E*. *coli* (dark grey columns) injections (mean number of cell per larva ± SEM, Mann-Whitney test, two-tailed, **p<0.005, ***p<0.001, *N*_*larvae*_ = 7–16 per condition, from two independent experiments). (E, F) *E*. *coli* log counts (CFU) in CTRL (E) and *csf3r* morphants (F) (mean number of CFU per larva ± SEM, Mann-Whitney test, two-tailed, ***p<0.001, *N*_*larvae*_ = 3–4 per condition). (G) Survival curve of *MO csf3r* and *MO CTRL* larvae infected with *E*. *coli* from 0 to 3 dpi (*N*_*larvae*_ is indicated in the figure, log rank test, *p<0*.*001*, from two independent experiments).

To investigate the role of neutrophils in bacterial clearance, we ablated neutrophils by two independent approaches. First, we specifically inhibited neutrophil development and function by knocking down the G-CSF/GCSFR pathway using a morpholino oligonucleotide (MO) specifically blocking *gcsfr/csf3r* translation (MO *csf3r*) [25,26]. Injection of MO *csf3r* in the neutrophil reporter embryos, *tg(mpx*:*GFP)*, led to approximately 70% reduction in the total number of neutrophils as compared to larvae injected with a control morpholino (MO CTRL) at 3 dpf (Fig 2A, 2C and 2D). We infected these morphants with 2500 CFUs fluorescent *E*. *coli*. Bacteria disappeared in the control larvae (Fig 2B and 2E) while they proliferated in neutrophil-depleted embryos (Fig 2B and 2F). The bacterial proliferation correlated with a further dramatic reduction in neutrophil number at 1 and 2 dpi (days post infection), suggesting neutrophil death (Fig 2D). Subsequently, infected *csf3r* morphants died between 2 and 3 dpi (Fig 2G) with overwhelming bacterial proliferation and neutropenia (S3B Fig).

**Fig 3:**
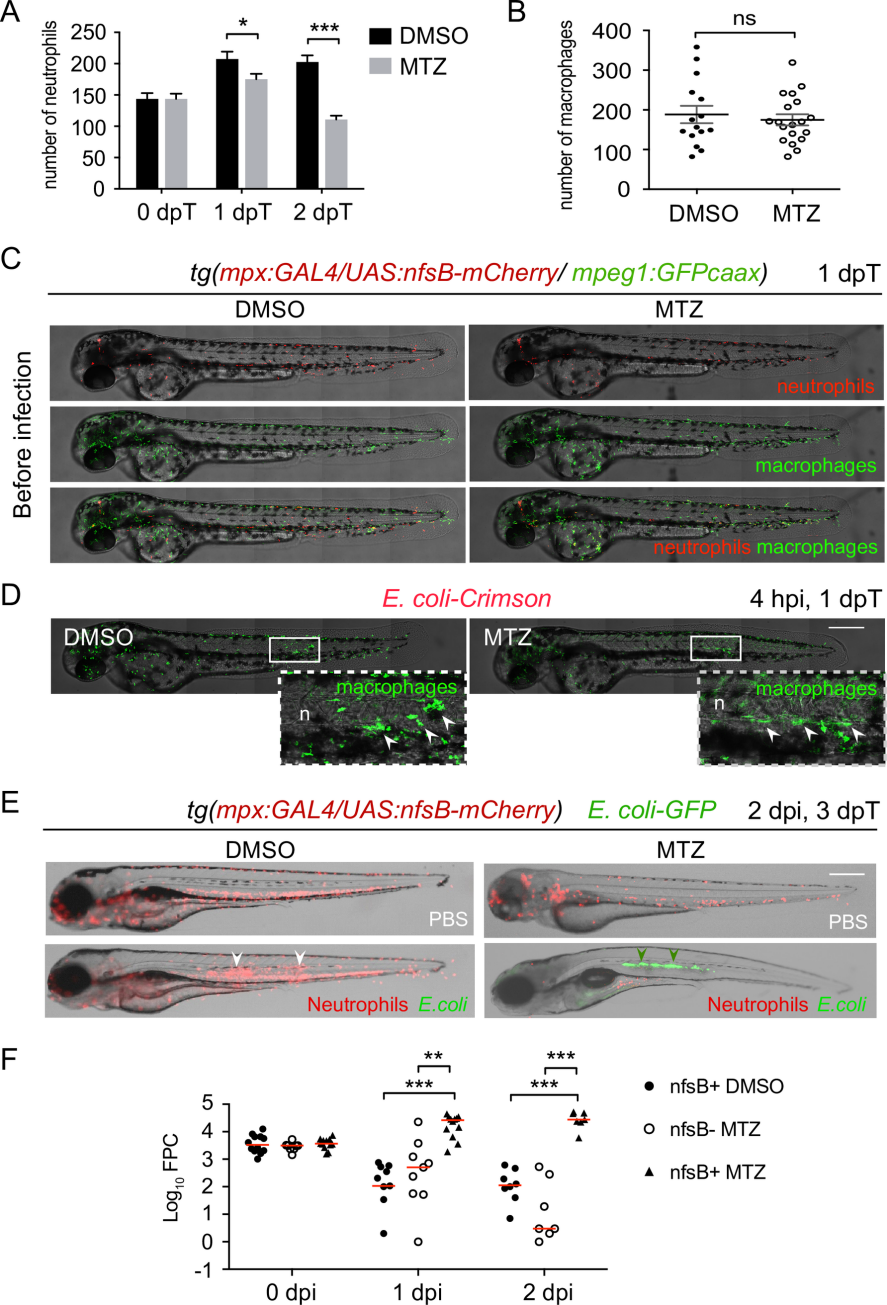
Neutrophil ablation using Nitroreductase/Metronidazole system leads to bacterial growth during notochord infection. (A-B-C-D) *Tg(mpx*:*Gal4/UAS*:*nfsB-mCherry/mpeg1*:*GFPcaax)* embryos were treated with DMSO or MTZ at 40 hpf and imaged at 0, 1 and 2 days post-treatment (dpT) with fluorescence microscopy. (A) Quantification of total neutrophils in DMSO and MTZ treated larvae at 0 and 1 and 2 dpT (mean number of neutrophils per larva ± SEM, Student’s test, one-tailed, *p<0.*05*, ***p<0.*001, N*_*DMSO*_ = 21, *N*_*MTZ*_ = 13–23). (B) Quantification of total macrophages in DMSO and MTZ treated larvae at 1 dpT (horizontal lines indicate mean values ± SEM, two independent experiments, Student’s test, two-tailed, ns: not significant, *p>0*.*05*, *N*_*DMSO*_ = 15, *N*_*MTZ*_ = 19). (C-D) Transgenic embryos were infected with *E*. *coli*-crimson in the notochord one day after MTZ treatment and imaged (C) before infection and (D) at 4 hpi with Spinning Disk confocal microscopy. (C) Representative overlay of maximum projections of montage acquisitions (mCherry and GFPcaax) with transmitted light images show neutrophil and macrophage distribution in DMSO and MTZ treated larvae before infection and (D) macrophage recruitment (arrowheads) at 4 hpi to the notochord (n). White boxes are zoomed areas. Similar results were obtained with 5 and 10 mM MTZ. (E) *Tg(mpx*:*Gal4/UAS*:*nfsB-mCherry)* embryos were treated with MTZ at 40 hpf and, at 3 dpf, larvae were injected either with PBS or *E*. *coli*-GFP in the notochord. The outcome of the infection was analysed by fluorescent microscopy. Larva images are representative overlays of fluorescence and transmitted light images at 2 dpi. In the absence of MTZ, neutrophils are massively recruited to the notochord and *E*. *coli* is cleared (white arrowheads). In MTZ-treated larvae, *E*. *coli* (green arrowheads) grow heavily. Scale bars: 400 μm. (F) Bacterial load quantification by Fluorescent Pixel Count (FPC) in MTZ treated *Tg(mpx*:*Gal4/UAS*:*nfsB-mCherry)* (nfsB^+^ MTZ) at 0, 1 and 2 dpi showing significant differences in the bacterial load with control groups (*Tg(mpx*:*Gal4/UAS*:*nfsB-mCherry)* treated with DMSO referred as nfsB^+^ DMSO and non transgenic siblings treated with MTZ referred as nfsB^-^ MTZ) (horizontal bars indicate the median, Kruskall-Wallis test with Dunn’s post-test, **p<0.01, ***p<0.001, *N*_*nfsB+ DMSO*_ = 9–12, *N*_*nfsB- MTZ*_ *=* 8–9, *N*_*nfsB+ MTZ*_ *=* 7–12).

We also ablated neutrophils, using *tg(mpx*:*Gal4/UAS*:*nfsB-mCherry)* embryos in which the *myeloperoxidase* promoter (*mpx*) indirectly drives the expression of nitroreductase in neutrophils. Treatment of *tg(mpx*:*Gal4/UAS*:*nfsB-mCherry)* embryos with metronidazole at 40 hpf specifically depleted neutrophils at 1 and 2 days post-treatment (Fig 3A). Since macrophages are required to clear apoptotic cells, we asked whether neutrophil death in MTZ treatment alters macrophage number or distribution in the triple transgenic line *tg(mpx*:*Gal4/UAS*:*nfsB-mCherry/ mpeg1*:*GFPcaax)*. At 1 dpT, MTZ treatment did not affect the number of macrophages and they were similarly distributed throughout the larva to the control (Fig 3B and 3C). Larvae were then infected with *E*. *coli*-crimson and 4 hours after *E*. *coli* injection, macrophages were recruited to the infected notochord in both MTZ and DMSO conditions, showing that ablation of neutrophil using *nfsB*/MTZ system does not impair macrophage response (Fig 3D). Infection outcome was then analysed in *tg(mpx*:*Gal4/UAS*:*nfsB-mCherry)* larvae infected with fluorescent *E*. *coli*-GFP. Similarly to *csf3r* morphants, bacteria were cleared in control larvae (*nfsB*^*+*^ DMSO and *nfsB*^*-*^ MTZ), while bacteria proliferated in embryos with low neutrophil density (*nfsB*^*+*^ MTZ), as shown by fluorescent microscopy and by quantification of bacterial burden (Fig 3E and 3F). These experiments demonstrate that neutrophils are essential for the control of notochord infection by *E*. *coli*.

We further investigated the relationship between neutrophil supply and bacterial disappearance in the notochord. Normal neutrophil levels were able to eliminate small amounts of bacteria (S3A Fig), but embryos with depressed neutrophil populations did not survive low bacterial loads (S3B Fig), while a higher bacterial inoculum overcame larvae with a normal neutrophil population (S3C Fig). However, by artificially increasing neutrophil density in the developing embryo through overexpression of *gcsfa*, we observed that increasing neutrophil density allow the embryo to cope with even higher amounts of injected bacteria (S3D Fig and S4A and S4C Fig). Similar results were observed by overexpressing *gcsfb* (S4 Fig). Our data reveals that the balance of neutrophils versus bacteria is instrumental for the outcome of the infection and that neutrophil populations are limiting in fighting the infection. To evaluate cell death, Sytox Green, a vital dye which labels DNA of dying cells, was injected into the vein of infected *tg(lyz*:*DsRed)* larvae. While PBS and low dose *E*. *coli* induced few cell death around the notochord, embryos experiencing neutropenia (i.e. infected with high dose *E*. *coli*) displayed increased cell death including dead neutrophils (S5 Fig). This suggests that when the neutrophil versus bacteria balance is not correct, neutrophils die by apoptosis. Of note, by contrast to neutrophil, macrophage number did not decrease, but instead increased 2 days after high dose infection (S6 Fig). These results are reminiscent to what happen in mammals in which neutrophil/bacteria ratio is fundamental for host defence [27].

### Neutrophil myeloperoxidase is not required to control notochord infection

**Fig 4:**
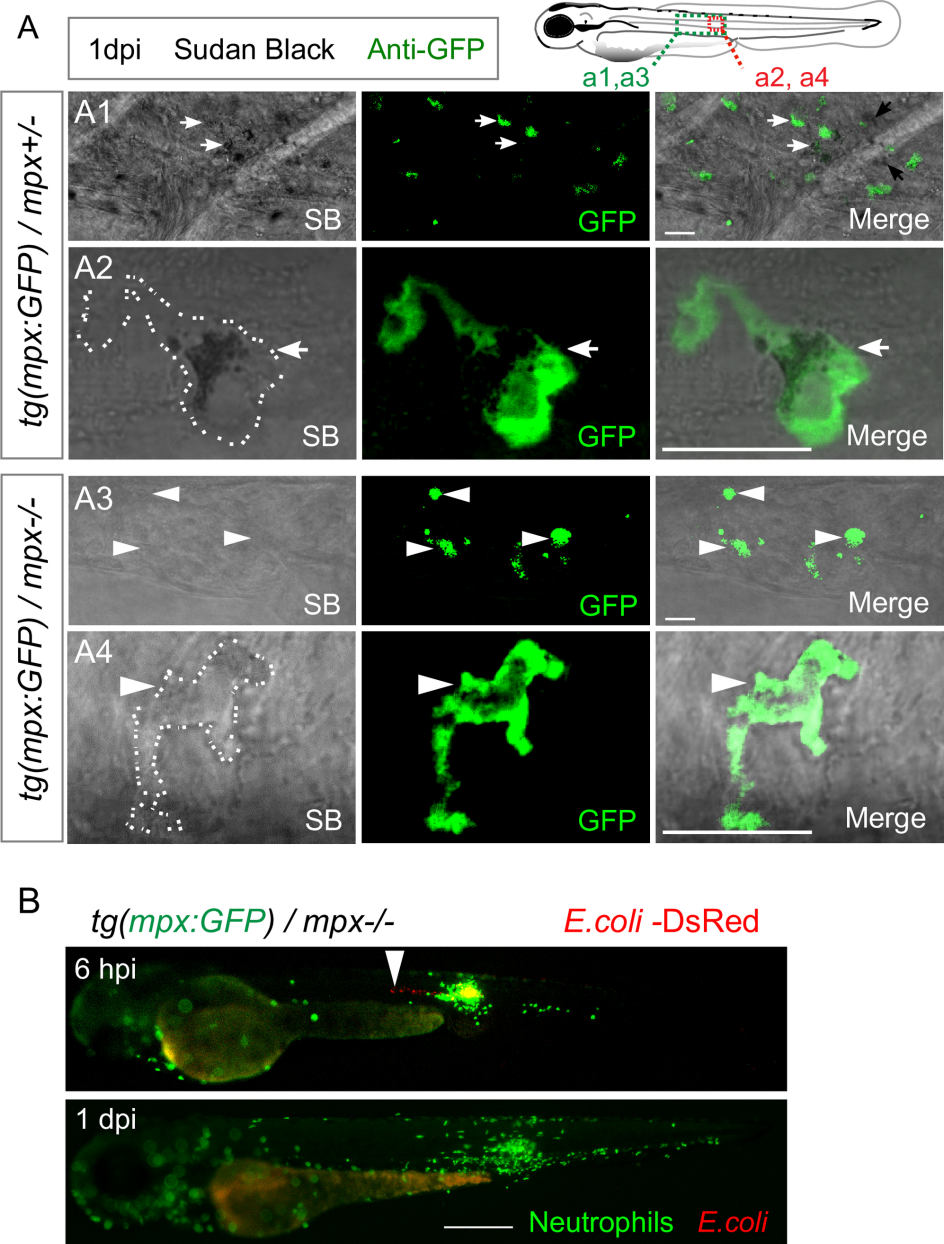
MPO is not required for bacterial clearance in the notochord. (A) *Tg(mpx*:*GFP)/mpx*^*+/-*^ (A1, A2) and *tg(mpx*:*GFP)/mpx*^*-/-*^ (A3, A4) embryos were infected with *E*. *coli* in the notochord. Sudan Black staining and immuno-detection of neutrophils (anti-GFP) were performed in whole embryos at 1 dpi. The top right panel shows the regions imaged by confocal microscopy in the larvae in A1 and A3 (green box) and in A2 and A4 (red box). Representative transmitted light images, overlaid with a maximal projection of confocal fluorescence images show the presence of black granules in the neutrophils (white arrows) of *tg(mpx*:*GFP)/mpx*^*+/-*^ embryos. MPX granules are absent in neutrophils (white arrowheads) of *tg(mpx*:*GFP)/mpx*^*-/-*^ embryos. Scale bars: 10 μm and white dotted lines outline neutrophils. (B) *Tg(mpx*:*GFP)/mpx*^*-/-*^ embryos were infected with *E*. *coli*-DsRed in the notochord. Neutrophils (GFP) and *E*. *coli* (DsRed) were imaged repeatedly in individual larvae using fluorescent microscopy at 6 hpi and 1 dpi. While *E*. *coli* locates in the notochord at 6 hpi (arrowheads), it disappears at 1 dpi. (*N*_*mpx+/-*_ *= 9*, *N*_*mpx-/-*_ *= 8* embryos per condition, from two independent experiments). Scale bar: 400 μm.

Our previous study revealed that approximately one-third of recruited neutrophils degranulate around infected notochords [21]. We therefore investigated the role of the neutrophil-specific myeloperoxidase (Mpx) that is present in the azurophilic granules, in bacterial clearance. We introduced the *mpx*:*GFP* transgene in the *mpx*-null mutant ‘spotless’ [28] to generate *tg(mpx*:*GFP)/mpx-/-* offspring in which neutrophils express the eGFP but lack Mpx activity. Active MPX in neutrophil granules can be visualized in zebrafish embryos using Sudan black staining [29]. Sudan Black staining confirmed that neutrophils did not carry Mpx activity in *tg(mpx*:*GFP)/mpx-/-* while in *tg(mpx*:*GFP)/mpx+/-* siblings, neutrophils contained active Mpx in their granules (Fig 4A). A low dose of fluorescent *E*. *coli* was injected in the notochord of 2 dpf *tg(mpx*:*GFP)/mpx-/-* embryos; neutrophils were normally recruited along the notochord, and the injected *E*. *coli* were cleared at 1 dpi as in the wild type (Fig 4B). Mpx is therefore not required for the clearance of *E*. *coli* in the notochord.

### Superoxide is produced in neutrophils of notochord-infected embryos

Neutrophils use different diffusible molecules to fight infections, including NO and ROS. We investigated NO production by neutrophils during the course of notochord infections using the NO reporter fluorescent probe DAF-FM-DA. We used *Salmonella* infected embryos as positive controls to detect NO production in neutrophils within the Aorta-Gonad-Mesonephros (AGM) (S7A Fig) [30]. As described [31], the notochord itself was labelled by DAF-FM-DA in uninfected embryos, but we could not observe any evidence of NO production by neutrophils in our notochord infection model (S7B Fig). L-NAME was previously shown to specifically inhibit NO synthases in zebrafish larvae [30]. To block NO production in our system, we thus treated larvae with L-NAME and injected *E*. *coli* into the notochord. We did not observe any difference in the outcome of the infection between L-NAME-treated larvae and controls (DMSO) (S7C Fig).

**Fig 5:**
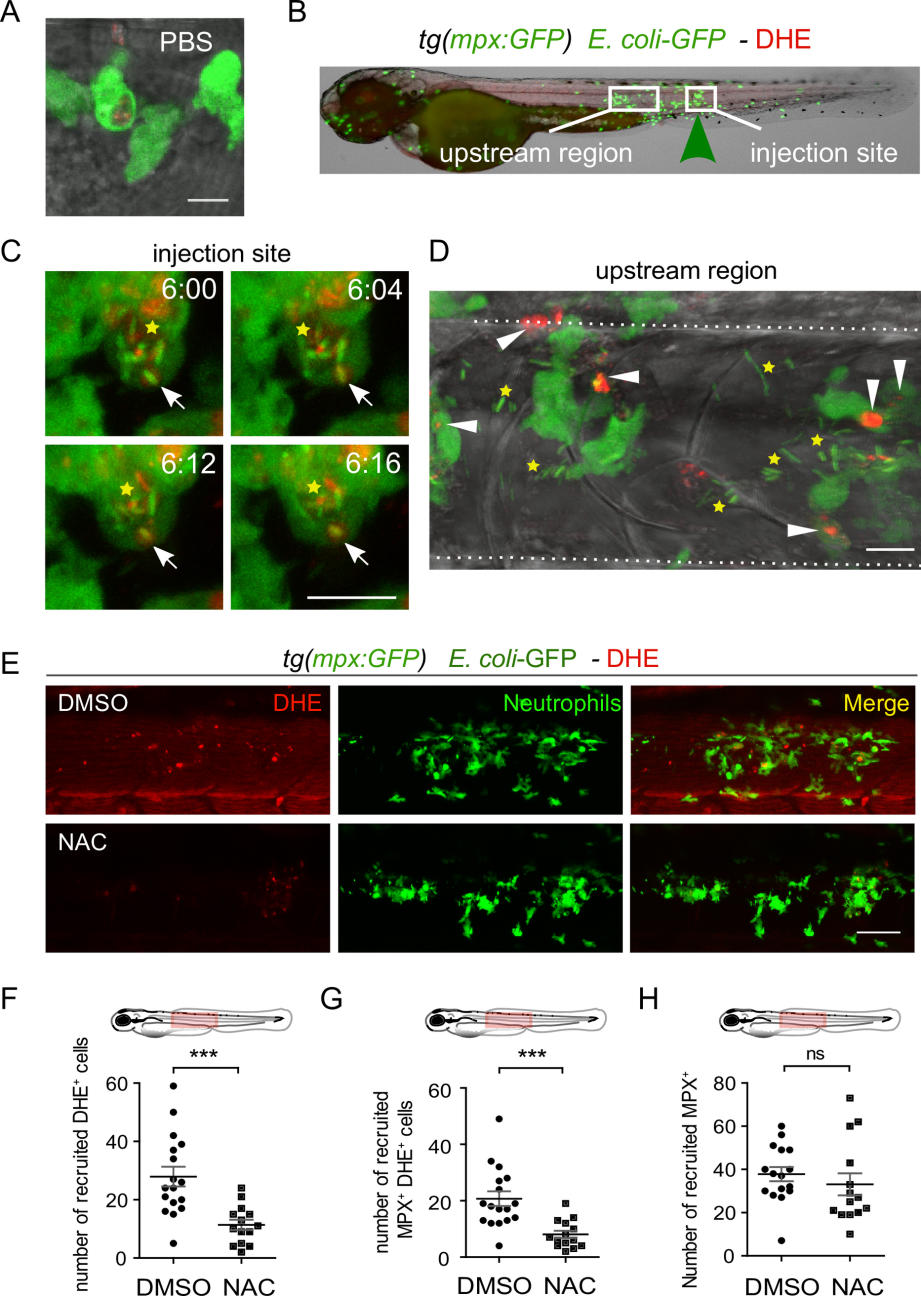
Superoxide is produced in neutrophils of infected larvae. (A-D) Two dpf *tg(mpx*:*GFP)* embryos were either injected with PBS (A) or infected with *E*. *coli*-GFP in the notochord (B, C, D). At 6 hpi, superoxide was detected in living animals using Dihydroethidium (DHE, red) and neutrophils were visualized using GFP fluorescence (green). (A) Representative transmitted light images, overlaid with a maximal projection of confocal fluorescence images show that superoxide is lightly produced in the recruited neutrophil at the injection site. (B) White boxes in the larva image show the regions imaged by high resolution confocal microscopy and green arrowhead shows the injection site. (C) Representative time-lapse maximum projections starting 6 hpi during 16 min, show superoxide presence in phagosomes (white arrows) bearing bacteria (yellow stars: *E*. *coli*-GFP, Green) in recruited neutrophils at the injection site. Time is in minutes. (D) Representative transmitted light images, overlaid with a maximum projection of confocal fluorescence images show superoxide in neutrophils (white arrowheads) over the *E*. *coli* (yellow stars) infected notochord. Scale bars: 15 μm, dotted lines encase the notochord (NC). (E) *Tg(mpx*:*GFP)* larvae were infected with *E*. *coli*-GFP in the notochord and treated either with DMSO or NAC. Trunk images are representative maximum projections of single fluorescence (DHE and GFP) and merge channels using confocal microscopy. Scale bar = 50 μm. (F-H) Quantification of recruited DHE^+^ cells (F), recruited DHE^+^ MPX^+^ cells (G), and recruited neutrophils (H) in indicated conditions (mean number of cell/larva ± SEM, ***p<0.*001*, ns: non significant, *N*_*DMSO*_ = 16–17 and *N*_*NAC*_ = 13–14, from three independent experiments). The diagrams represent the regions selected for the counting.

The phagocyte NADPH oxidase and ROS production play a key role in the elimination of engulfed bacteria [4]. To detect intracellular ROS accumulation in the form of superoxide anions in *tg(mpx*:*GFP)* embryos infected with *E*. *coli*, we used Dihydroethidium (DHE), a cell permeable probe that fluoresces in red after reacting with superoxide within the cell [32,33]. First, we imaged the injection site, where some bacteria initially leaked from the pierced notochord and got engulfed by neutrophils and observed that these phagocytosing leukocytes, abundantly produced superoxide in intracellular compartments harboring bacteria, which are most probably phagosomes (Fig 5A and 5B). Green fluorescent *E*. *coli* were rapidly lysed within 20 minutes in the putative phagosome (Fig 5B and 5C and S1 Video). We then imaged the upstream region, where bacteria are separated from the recruited neutrophils by the notochord collagen sheath. Interestingly, these recruited neutrophils also produced large amounts of superoxide, even though they had not phagocytosed bacteria (Fig 5D). DHE was also detected at a basal level in notochord surrounding tissues (Fig 5E). To test the specificity of DHE staining in detecting superoxide anions we treated infected embryos with N-acetyl-cysteine (NAC), a broad-specificity ROS scavenger. We observed a general decrease of DHE staining within cells of the trunk and more particularly a decrease of DHE^+^ recruited cells (Fig 5E and 5F) and of DHE^+^ recruited neutrophils (Fig 5E and 5G) around the infected notochord while the number of recruited neutrophils was unchanged by the treatment (Fig 5E and 5H), confirming that DHE probe specifically detects ROS in this model.

### NADPH oxidase activity is essential for bacterial killing at a distance and larva survival to notochord infection

**Fig 6:**
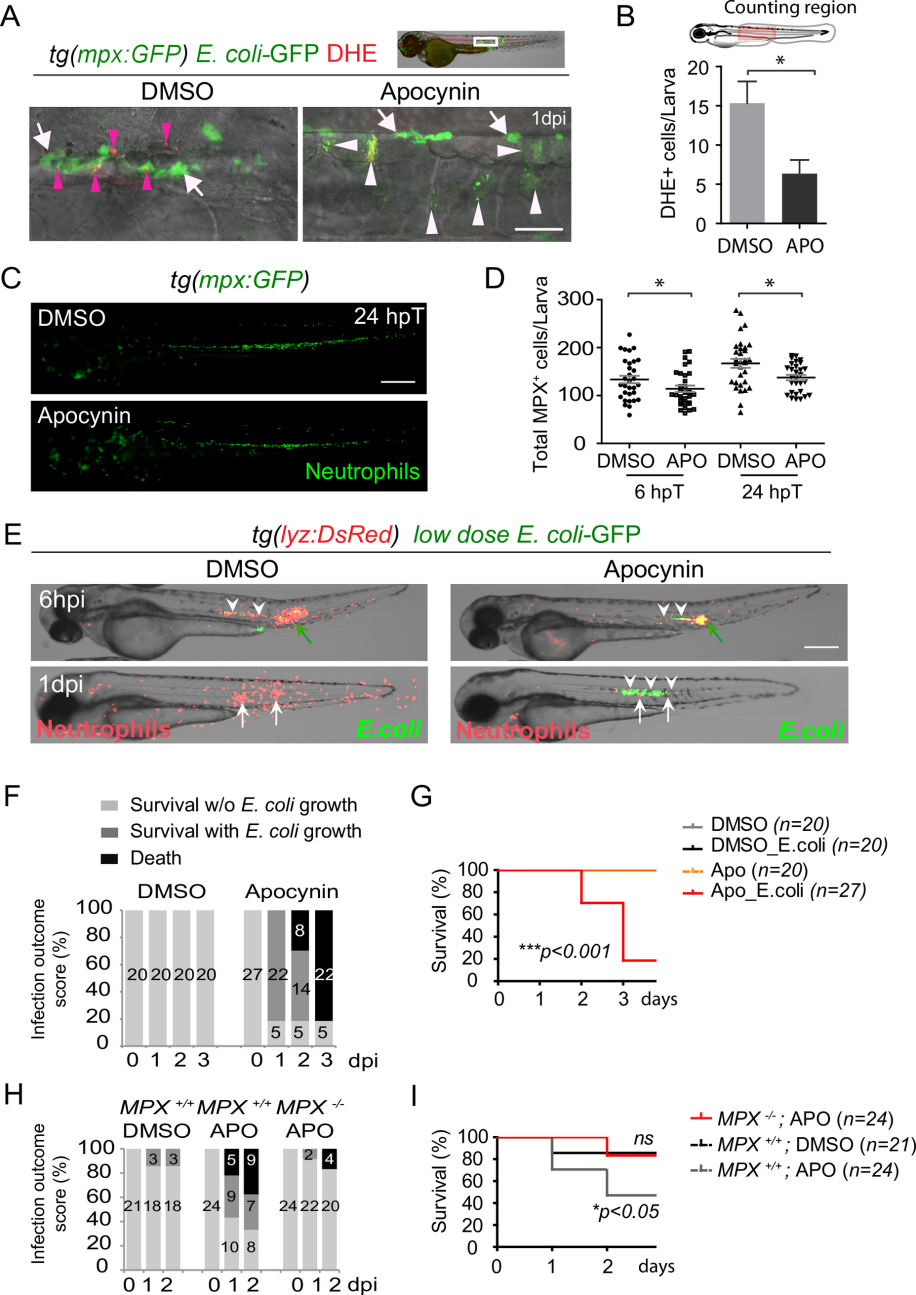
NADPH oxidase inhibitor Apocynin increases susceptibility to notochord infection. (A-B) *E*. *coli-*GFP were injected in the notochord of 2 dpf *tg(mpx*:*GFP)* embryos in DMSO or Apocynin treatment conditions. (A) At 1 dpi, superoxide production was visualised using DHE (red), neutrophils and *E*. *coli* were detected using GFP. Notochord images are representative maximum projection of fluorescence confocal images overlaid with transmitted light images. Pink arrowheads show DHE^+^ neutrophils, white arrows show DHE^-^ neutrophils and white arrowheads: *E*. *coli*, scale bars: 30 μm. (B) Quantification of DHE-positive cells in DMSO and Apocynin treated larvae (mean ±SEM, *N*_*larvae*_ = 5 per condition, Mann-Whitney test, one-tailed, * p<0.05). (C, D) T*g(mpx*:*GFP)* embryos were treated with Apocynin (APO) or DMSO at 2 dpf. Neutrophils (GFP) were imaged using fluorescent microscopy at 6 hours post-treatment (hpT) and 24 hpT. (C) Representative fluorescent images of Apocynin or DMSO treated larvae at 24 hpT. Scale bar: 400 μm. (D) Corresponding counts of total neutrophil population in indicated conditions (mean ± SEM, *N*_*DMSO*_ = 31 and *N*_*APO*_ = 29, Mann-Whitney test, two-tailed, * p<0.05, representative of 2 independent experiments). (E, F, G) Two dpf *tg(lyz*:*DsRed)* embryos were infected in the notochord with *E*.*coli*-GFP and treated with Apocynin. (E) Neutrophils (DsRed) and *E*. *coli* (GFP) were imaged repeatedly in individual larvae using fluorescent microscopy at 6 hpi and 1 dpi. Bacteria (white arrowheads) were present at 6 hpi in both DMSO- and Apocynin-treated embryos. At 1 dpi, bacteria disappeared in DMSO-treated embryos (arrows) while their number increased in Apocynin-treated embryos (white arrowheads). (F) Infection outcome of *E*. *coli* infected embryos after in DMSO or Apocynin treatments were scored from 0 to 3 dpi (the number of larvae is indicated in the columns). (G) Survival curves of larvae uninfected and infected with *E*. *coli* from 0 to 3 dpi in DMSO or Apocynin treatments. (*N*_*larvae*_ is indicated in the figure, log rank test, p<0.001, from two independent experiments). (H) Two dpf *mpx*^*+/+*^ *or mpx*^*-/-*^ embryos were infected in the notochord with *E*. *coli*-GFP and treated either with DMSO or Apocynin (APO). Infection outcome of *E*. *coli* infected embryos were scored from 0 to 2 dpi (the absolute number of larvae is indicated in the columns). (I) Survival curves of *mpx*^*+/+*^ *or mpx*^*-/-*^ larvae infected with *E*. *coli* from 0 to 2 dpi in DMSO or Apocynin treatments (*N*_*larvae*_ is indicated in the figure, log rank test, *p<0*.*01*, from two independent experiments).

To investigate whether this superoxide production could be involved in bacterial killing, we used Apocynin, a NADPH oxidase (NOX) inhibitor [34,35]. Upon notochord infection, Apocynin-treated embryos had reduced number of superoxide producing cells, including recruited DHE^+^ neutrophils at the inflammation site, as compared to DMSO-treated larvae (Fig 6A and 6B), showing the efficiency of Apocynin as a NOX inhibitor in zebrafish. To test whether Apocynin alters the steady state of neutrophils, *tg(mpx*:*GFP)* larvae were treated with this drug at 2 dpf. Apocynin treatment decreased the total number of neutrophils after 6 or 24 h of treatment, but by less than 15% (Fig 6C and 6D), showing that this approach is suitable to test the role of NOX in zebrafish neutrophils. Therefore, we infected *tg(lyz*:*DsRed)* embryos with a very low dose of *E*. *coli* (<1000 CFUs) in the notochord. Even with the very low dose infection, 80% of Apocynin-treated embryos failed to clear the bacteria, while all bacteria were efficiently killed in DMSO-control embryos (Fig 6E). Apocynin-treated embryos displayed unrestricted bacterial growth in the notochord at 1 dpi, as demonstrated with fluorescence microscopy (Fig 6E and 6F). This was correlated with neutropenia and eventually death at 2–3 dpi (Fig 6F and 6G). The effect was specific to the clearance of bacteria in this notochord infection model since Apocynin treatment did not interfere with the clearance of bacteria injected in the muscle, where phagocytosis occurs (S8 Fig). Similar results were obtained using another NOX inhibitor [36], VAS2870 (VAS) (S9 Fig).

Interestingly, in mammals, Apocynin activity requires that target cells do express an active Mpx [35]. Therefore, we compared the results of Apocynin treatment in *mpx*^*-/-*^ and *mpx*^*+/+*^ infected embryos, and observed that Apocynin increased susceptibility to notochord infection only in the presence of Mpx (Fig 6H and 6I). Thus, Apocynin action is also dependent on Mpx in zebrafish, and thus specifically acts on neutrophils. Overall, these data thus strongly suggest that inhibition of superoxide production in neutrophils increases susceptibility to notochord infection.

**Fig 7:**
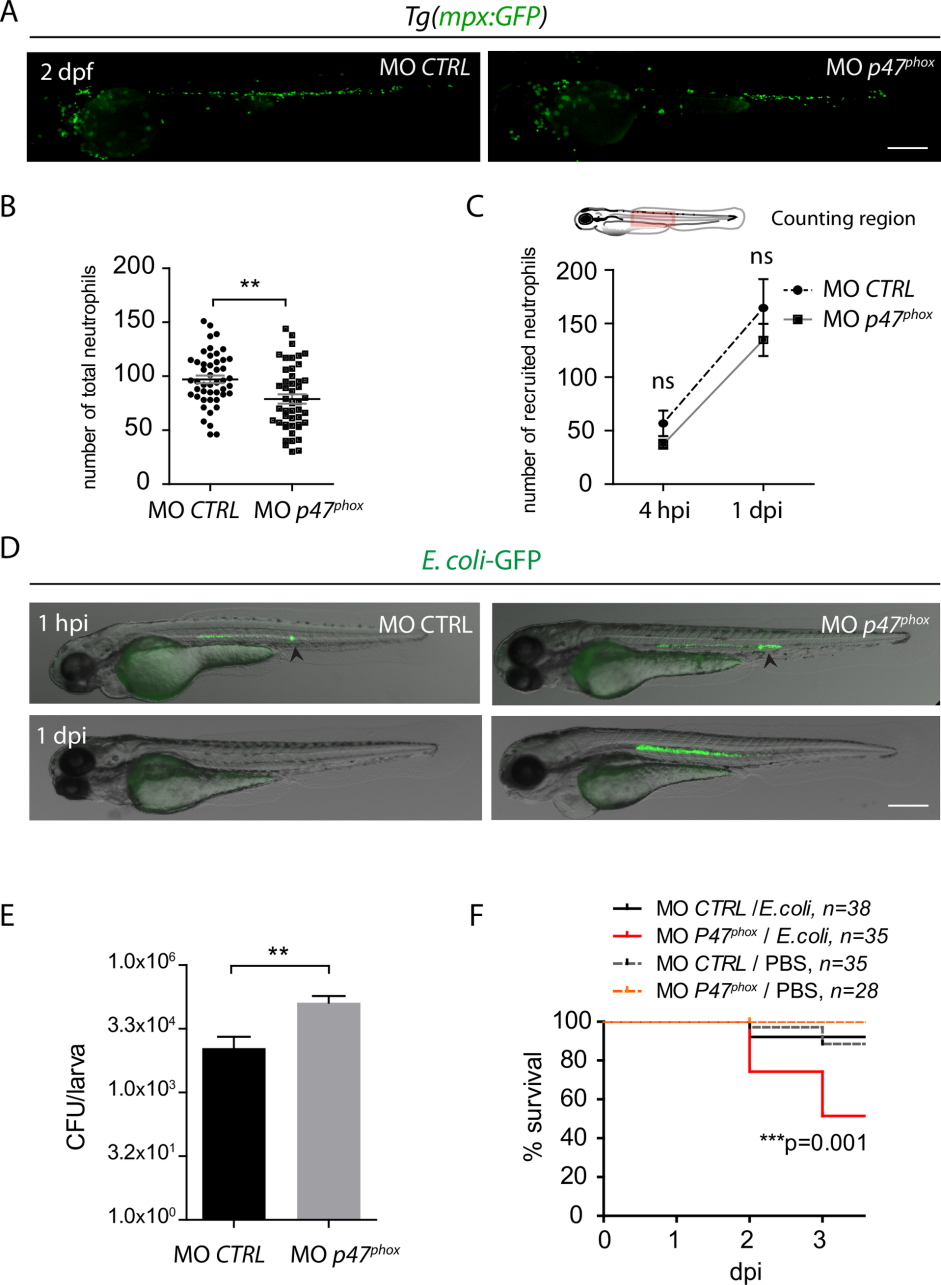
*p47*^*phox*^ is required for bacterial clearance at a distance and host survival following notochord infection. (A-C) *Tg(mpx*:*GFP)* embryos were injected at the one cell stage with either *p47*^*phox*^ morpholino (MO *p47*^*phox*^) or a control morpholino (MO CTRL). Steady-state neutrophil populations were imaged in 2 dpf morphants using fluorescence microscopy. Scale bar: 400 μm. (B) Neutrophil counts in whole larvae (mean number of neutrophils per larva ± SEM, *N*_*MO CTRL*_ = 47 and *N*_*MO P47*_ = 47, Mann-Whitney test, two-tailed, **p<0.*005*, from three independent experiments). (C) At 2 dpf, *p47*^*phox*^ and CTRL morphants were infected with *E*. *coli*-GFP in the notochord and imaged using fluorescence microscopy at 4 hpi and 1 dpi. Graph represents mean number of recruited neutrophils per larva ± SEM in the notochord region (*N*_*MO CTRL*_ = 10–14 and *N*_*MO P47*_ = 12–16, Mann-Whitney test, one-tailed, *p>0*.*05* ns: non significant, from two independent experiments). (D) *p47*^*phox*^ and CTRL morphants were infected with *E*. *coli*-GFP in the notochord at 2 dpf and GFP fluorescence (bacteria) was imaged repeatedly in individual larva, fluorescence was overlaid with transmitted light images at 1 hpi and 1 dpi. Black arrowheads indicate the infection site, scale bar: 400 μm *(N*_*MO CTRL*_ *= 19/21 and N*_*MO p47*_ *= 16/21)*. (E) The CFU counts at 1 dpi in notochord infected of *p47*^*phox*^ and CTRL morphants (mean number of CFU per larva ± SEM, *N*_*MO CTRL*_ = 13 and *N*_*MO P47*_ = 8, Mann-Whitney test, one-tailed, **p<0.*01*, from three independent experiments). (F) Survival curves of *p47*^*phox*^ and CTRL morphants that have been injected with PBS or *E*. *coli* in the notochord from 0 to 3 dpi (*N* is indicated in the figure, log rank test, ***p<0.*001*, from 4 independent experiments).

To further examine the role of phagocyte NOX, morpholino-mediated gene knockdown was used. Injection of *p47^phox^* MO in *tg(mpx*:*GFP)* did not induce noticeable morphological defects, but, as expected, decreased superoxide production in neutrophils following infection compared to control morpholino (*CTRL* MO) (S10 Fig). To address the effect *p47^phox^* MO on the development and the recruitment of neutrophil, we analyzed *tg(mpx*:*GFP) p47^phox^* morphants before and after *E*. *coli* infection in the notochord at 2 dpf. Although *p47^phox^* morphants displayed 20% less neutrophils than in control morphants, (Fig 7A and 7B) these leukocytes were recruited in normal numbers to the notochord at 4 hpi and 1 dpi (Fig 7C), showing that *p47^phox^* morphants can mobilize neutrophils properly during the infection. Then, *p47^phox^* morphants were infected in the notochord with *E*. *coli*-GFP. *P47^phox^* MO induced higher bacterial burden as evidenced by fluorescence microscopy (Fig 7D) and CFUs counts (Fig 7E). This was correlated with an increase in the severity of infection (Fig 7F).

**Fig 8:**
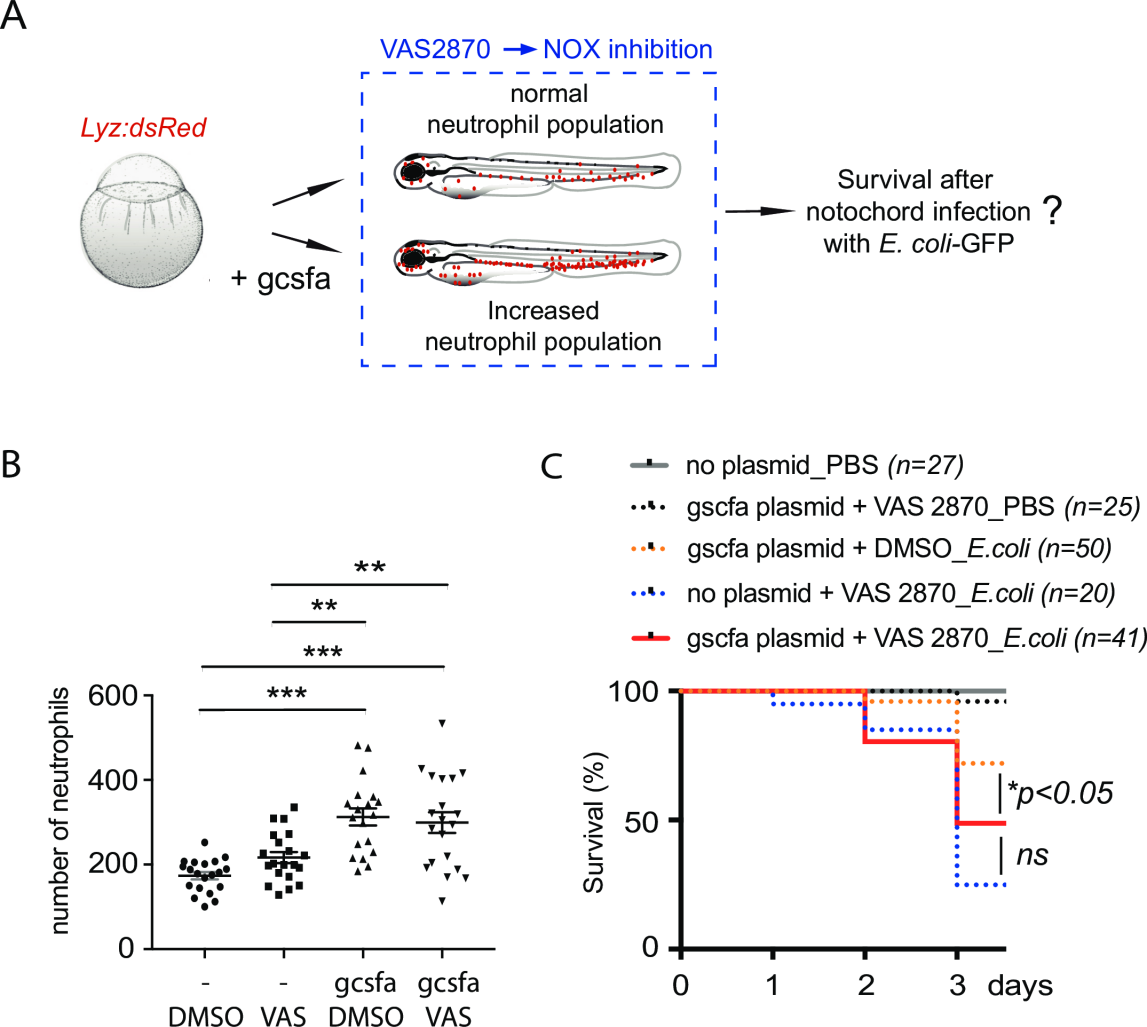
*gcsfa* overexpression does not improve the survival of NOX incompetent larvae during notochord infection. (A) Diagram shows the experimental strategy to induce high neutrophil number during NOX inhibition in zebrafish larvae. To increase neutrophil density, *tg(lyz*:*DsRed)* embryos were injected with the *gcsfa* over-expressing plasmid at one cell stage. At 2 dpf, larvae were treated with VAS2870 to inhibit NOX enzyme. Then, NOX incompetent larvae were infected with *E*. *coli*-GFP for monitoring of the survival. (B) *Tg(lyz*:*DsRed)* larvae were imaged 5 h after treatment with DMSO or VAS2870 using fluorescence microscopy. The plot shows quantification of total neutrophils in indicated conditions (horizontal lines indicate mean number of neutrophils ± SEM, from two independent experiments, ANOVA with Tukeys’ post-test, **p<0.01 and ***p<0.*001*). (C) One hour after treatment (at 2 dpf) larvae were infected in the notochord with *E*. *coli*-GFP. Survival curves of larvae in indicated conditions from 0 to 3 dpi (*N*_*larvae*_ is indicated in the figure, log rank test, *p>0.*05*, ns: not significant).

As neutrophils are instrumental for larva survival and bacterial clearance during notochord infection and as pharmacological (apocynin and VAS2870) and genetic (*p47*^*pho*x^ morpholino) inhibition caused a slight decrease of neutrophil numbers, we tested whether inducing high neutrophil number in the context of NADPH incompetence could restore survival of the infected larvae. One-cell stage *tg(lyz*:*DsRed)* embryos were thus injected with *gcsfa* expressing plasmid and 2 days later were treated either with DMSO or VAS2870 (Fig 8A). Beside the fact that *gcsfa* forced expression increased the number of neutrophils compared to controls (Fig 8B), it did not restore a better survival of the infected larvae in the presence of Nox inhibitor VAS2870 (Fig 8C).

Altogether these data show that NOX-induced superoxide is necessary for bacteria elimination at a distance by neutrophils.

## Discussion

Many studies have used the zebrafish embryo model to address the respective roles of neutrophils and macrophages in eliminating invading bacteria, but in all instances, at least one of these two cellular populations had direct access to the bacteria. In our model neither neutrophils nor macrophages could reach the bacteria. We first observed an active recruitment of both macrophages and neutrophils around the infected notochord that is correlated with the elimination of the bacteria in the notochord within 24 hours. Specifically depleting individual myeloid populations, we have investigated their contribution in the clearance of *E*. *coli* at a distance and describe molecular pathways involved in bacterial elimination by neutrophils.

Using chemical and genetic ablation of macrophages, we revealed that despite being massively recruited to the notochord, macrophages are not required for the bacterial killing. By contrast, whichever the strategy to lower the amount of neutrophils within the developing zebrafish, the embryo becomes unable to cope even with low-dose infection, leading to bacterial proliferation and death of the embryo, showing that neutrophils are essential to control notochord infection. Further analysis should reveal whether other mechanisms are also involved in the death of *E*. *coli* within the notochord, such as complement-mediated killing or killing by the notochordal cells.

Furthermore, we highlight the importance of the numerical balance between neutrophils and bacteria to the outcome of notochord infection in which phagocytosis is not feasible. This observation suggests that the bactericidal molecules produced by the neutrophils to fight the bacteria are produced in limiting quantities. During *Salmonella* infections, the correct population of neutrophils is maintained through a mechanism of demand-driven granulopoiesis in the main site of hematopoietic stem cells emergence, i.e., the AGM [30]. Similarly, we observed here, that in low dose *E*. *coli* infections, the host is able to increase the neutrophil pool to control notochord infection. However, too low a neutrophil/bacteria ratio (either by increasing bacterial load or decreasing the number of neutrophils) results in bacterial proliferation, onset of neutropenia, and death within 2 to 3 dpi. Conversely, the neutrophil-enriched embryos can cope with a very high dose of bacteria. These data are reminiscent of results in human where the maintenance of a proper pool of neutrophil is critical for effective bacterial killing [27,37,38], emphasizing thus the relevance of the tractable zebrafish larvae system for the study of dynamic interactions between neutrophil bactericidal activity and bacteria in vivo.

To capture and kill microbes they cannot phagocytize, neutrophils have been described to expel their chromatin to form Neutrophil Extracellular Traps (NETs), but this may lead to neutrophil death (Netosis) [39,40]. NET formation relies on complex intracellular processes involving the activity, among others, of myeloperoxidase [41]. We report here that myeloperoxidase activity is not necessary to fight the infection in our experimental system. This shows that MPX dependent-NET formation is not responsible for bacterial killing at a distance. However Myeloperoxidase may not be required with all stimuli, since MPO was shown to be dispensable for NET induction in infections with *Pseudomonas aeruginosa* or *Staphylococcus aureus*. Therefore, we cannot exclude the involvement of MPO-independent NETs in our system [42].

**Fig 9:**
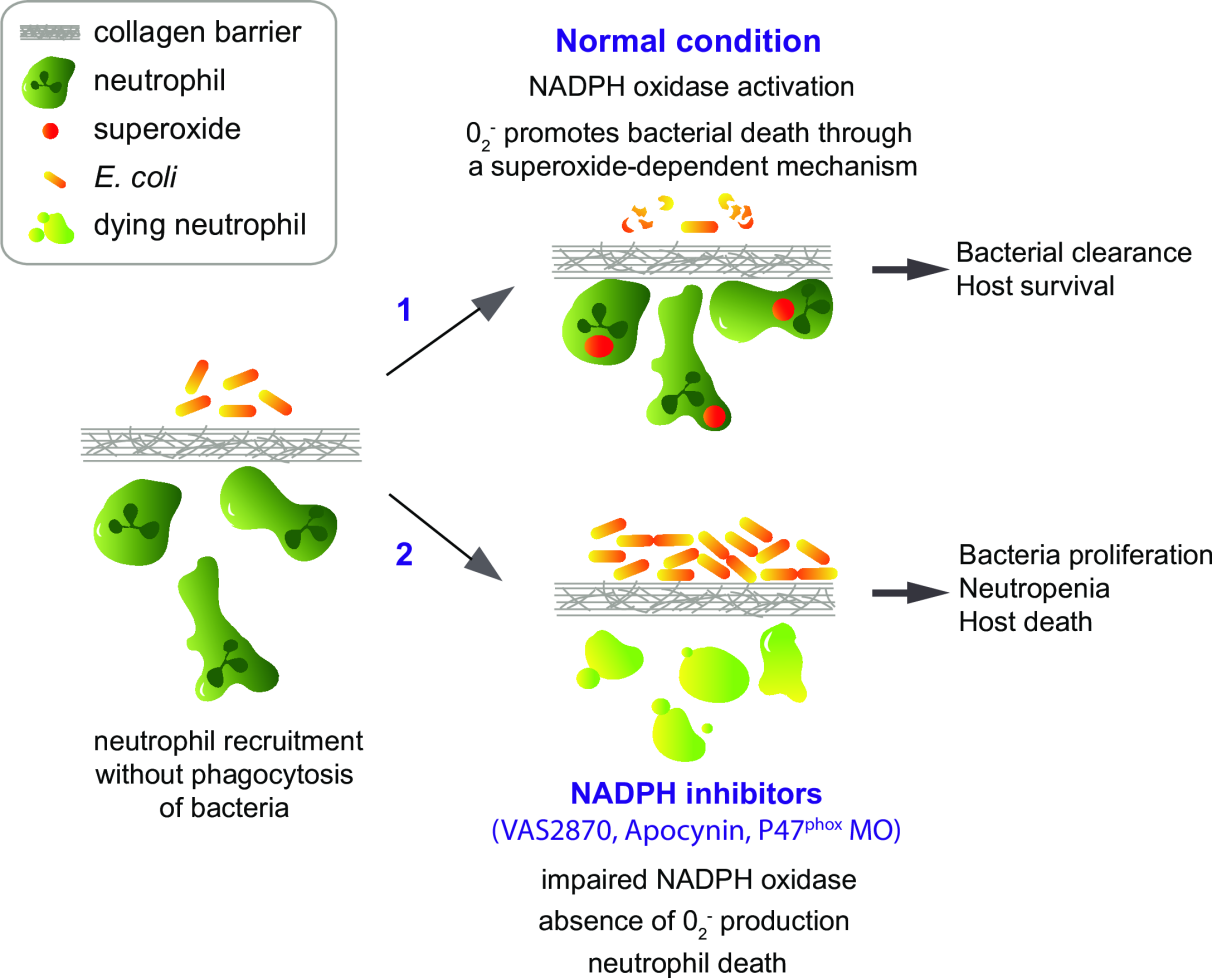
Graphical abstract of neutrophil defence against distant bacteria during notochord infection. Low dose of *E*. *coli* infection in the notochord leads to the rapid recruitment of neutrophils to the notochord. During the first phase of infection, neutrophils cannot penetrate the collagen sheath and engulf bacteria. 1/ In normal condition, NOX activity in recruited neutrophils leads to the production of the ROS superoxide. Superoxide production participates in bacterial clearance without neutrophil-microbe physical contact through a yet unknown mechanism and results in host survival. 2/ Reducing the ROS superoxide using a drug that inhibits NADPH Oxidase assembly (VAS2870) or a drug that blocks NADPH Oxidase in the leukocytes (Apocynin) or using a *p47*^*phox*^ morpholino results in bacteria growth in the notochord and host neutropenia and death.

We report here that NOX activity and the production of superoxide by neutrophils are essential to cope with notochord infection by *E*. *coli*. Indeed, using fluorescent probes, we showed that neutrophils swarm around the notochord and produce large amounts of superoxide. Treatments of the embryos with inhibitors of NOX assembly, VAS2870 and Apocynin, or the specific knock down of Nox subunit p47^phox^ using morpholinos, lead to bacterial proliferation and increased severity of the infection. This is accompanied with the decrease of superoxide production in neutrophils, consistent with an essential role of superoxide in the clearance of *E*. *coli* without direct phagocytosis (Fig 9). Apocynin activity was shown to be dependent on the presence of myeloperoxidase in neutrophils [35]. In our model, Apocynin has almost no activity in *mpx*^*-/-*^ mutant, reinforcing the specificity of its effect. This demonstrates that Nox activity in neutrophils is required for bacterial clearance in the notochord.

The present work raises different questions related to the death of the different actors, the bacteria, the neutrophils, and the embryo. Foremost is the question as to how bacteria are killed at a distance by neutrophils. Neutrophils massively degranulate around the infected notochord [21] and we show here that an oxidative burst is necessary for bacterial elimination. Superoxide is known to be weakly bactericidal [4,43], but is rapidly converted to hydrogen peroxide by dismutation. Although products of NADPH oxidase are soluble, they are rapidly consumed by reactions with other targets within a limited diffusion distance [44]; however we cannot exclude the possibility that these ROS diffuse through the very thin (<1 μm) collagen sheath. A more possible scenario, would be that superoxide is not involved in a direct killing mechanism but instead is interacting with a host- or microbe- derived species, triggering a superoxide-dependent process (Fig 9). Indeed, besides inducing oxidative stress, ROS also serve as signalling molecules to regulate biological processes. One of the best-understood mechanism of redox signalling involves H_2_O_2_-mediated oxidation of cysteine residues within proteins, altering thus their function [45]. These reversible modifications could trigger activation of signalling cascade and the release of bactericidal agents. Another important target of ROS is the transcription factor NF-κB which is known to control many aspects of the immune response [46]. Therefore neutrophil superoxide may act as a second messenger of a killing strategy at a distance. Why do neutrophils die when the bacteria/neutrophil ratio is too high in favor of the invaders? If bacteria proliferate within the infected notochord, then neutrophils massively die, and the embryo becomes neutropenic. This could be due to a factor released by the densely packed bacteria within the notochord. However, there may be no reason why this virulence factor would specifically kill neutrophils while sparing the highly endocytic macrophages that are also massively recruited to the notochord but not affected by bacterial proliferation. For this reason, we propose that death of neutrophils could rather be a consequence of the excessive concentration of bacteria-derived molecules, similarly to a quorum sensing mechanism, triggering hyper activation of the neutrophils and leading to their death [47]. This hyper activation, akin to a local cytokine storm is likely also responsible for the death of the embryo in cases where *E*. *coli* proliferates within the notochord. Importantly, we have no indication that the bacteria used in this study could kill the embryo by themselves. We consider that in cases where the embryos die, it is the consequence of their heavy inflammatory status mimicking a cytokine storm. This hypothesis is consistent with the similar outcome observed with pathogenic and non-pathogenic *E*. *coli* strains, as well as with our experiments with mycobacteria. We have demonstrated that mycobacteria can replicate within the notochord ultimately leading to notochord break down, without triggering the heavy inflammation described here with *E*. *coli*. The subsequent fate of the embryo depends on the virulence of the mycobacteria. The non-virulent *Mycobacterium smegmatis* is eliminated by phagocytosis, leading to the host survival while *M*. *marinum* resists destruction by phagocytosis and keeps proliferating until the host dies [48]. Conversely, *E*. *coli* only effectively kills infected embryos when injected alive in excessive amounts in the notochord where this triggers a heavy inflammation that kills the neutrophils and ultimately the embryo.

To overcome killing by neutrophils, some pathogenic bacteria developed strategies to avoid contact with phagocytes. Some pathogens invade tissues that are inaccessible to phagocytes, while other employ strategies to prevent engulfment [3]. They harbor on their surfaces molecules preventing recognition by phagocytes, such as capsular antigens O75 and K5 of uropathogenic *Escherichia coli* (Burns and Hull, 1999) and polysaccharide capsules of *Streptococcus pneumoniae* that increase the resistance to phagocytosis. *Staphylococcus aureus* secretes the 16 kD Extracellular fibrinogen binding protein that blocks its phagocytosis by human neutrophils by forming a “capsule”-like shield [49]. By contrast, *Yersinia pestis* (the agent of bubonic and pneumonic plaque), *Yersinia pseudotuberculosis* (gastroenteritis) and *Yersinia enterocolitica* (gastroenteritis and mesenteric adenitis) are able to inhibit the actin cytoskeleton required for engulfment, through the secretion of effector proteins into the cytoplasm of the immune cell, leading to decreased phagocytosis by neutrophils and increased virulence [3]. Oxidative burst at a distance might be an alternative mechanism employed by neutrophils to prevent such escape mechanisms. Further investigations should determine whether host targeted therapeutic strategies may be beneficial against medically relevant infections, especially in patients suffering from Chronic Granulomatous Disease whose neutrophil function is deficient for NADPH activity.

## Methods

### Ethics statement

Animal experimentation procedures were carried out according to the European Union guidelines for handling of laboratory animals (http://ec.europa.eu/environment/chemicals/lab_animals/home_en.htm) and were approved by the Comité d'Ethique pour l'Expérimentation Animale under reference CEEA-LR-13007 and APAFIS#5737–2016061511212601 v3. Fish husbandry and experiments were performed at the University of Montpellier. Embryos were obtained from the University of Montpellier and the Institut Pasteur. Experiments were performed on 0 hour to 5 days past fertilization stages when the embryos were used.

### Fish husbandry

Fish maintenance, staging and husbandry were performed as described [21] with golden strain and transgenic lines. *Tg(mpeg1*:*mCherry-F)ump2*, referred as *tg(mpeg1*:*mCherry-F)* [50], *tg(mpeg1*:*GFPcaax)* [51] *and tg(mfap4*:*mCherry-F) (ump6tg*, *present study)* were used to visualize macrophages. *Tg(mpx*:*GFP)i114* and *tg(lyz*:*DsRed)nz50* used to label neutrophils and the *mpx*^*t30963/t30963*^ *null* ‘spotless’ mutant, are referred here as *tg(mpx*:*GFP)* [52], *tg(lyz*:*DsRed)* [53] and *mpx*-/- [28], respectively. *Tg(rcn3*:*gal4) (PD1023)* crossed with *tg(UAS*:*mCherry) (PD1112)* were used to visualize notochordal cells [54]. *Tg(mpx*:*Gal4/UAS*:*nfsB-mCherry)* was used to ablate neutrophils [55]. *Tg(mpeg1*:*Gal4/UAS*:*nfsB-mCherry)* was used to ablate macrophages [26]. Embryos were obtained from pairs of adult fishes by natural spawning and raised at 28.5°C in tank water. Embryos and larvae were staged according to [56].

### Generation of the macrophage reporter line, Tg(mfap4:mCherry-F)

The Mfap4 promoter used to drive the specific expression of membrane-targeted mCherry in macrophages was amplified using the upstream primer zMfap4_3P1 (5’ ATC CAT GCC CTT CGA CTG TT 3’) and the zMfap4_123E2N primer matching the start of the second exon of the Mfap4 gene (5’ TAT AGC GGC CGC ACA GCA CGA TCT AAA GTC ATG AA 3’). The 2.4 kb amplified fragment was digested by NotI, and ligated to the coding phase of the farnesylated mCherry protein so that the Mfap4 AUG is in phase with the downstream mCherry-F ORF on a I-SceI meganuclease and Tol2-derived vector (GenBank accession no. GU394080). The resulting plasmid was injected, together with I-SceI meganuclease, into embryos at the one-cell stage.

### *E*. *coli* and *Salmonella* injections

*E*. *coli* K12 or *Salmonella enterica* serovar Typhimurium (here called *Salmonella*) carrying plasmids encoding GFP or DsRed fluorescent proteins were injected in the notochord of 2 dpf embryos as described [21]. Four different doses of *E*. *coli* were used: very low (1000 CFU), low (<3000 CFU), high (3000<n<6000 CFU) and very high (>7000 CFU). 3000 CFU of *Salmonella* were injected in the hindbrain or in the notochord. Enteroinvasive *E*. *coli* AIEC bacteria strain LF82 [57] and its mutant, LF82-ΔlpfA [58] and JM83ΔmsbB [59] were injected at a low dose (CFU<3000) in the notochord.

### Quantification of bacterial load by CFU counts and by Fluorescent Pixel Counts

CFU counts were performed as previously described [21]. For quantification of bacterial load by Fluorescent Pixel Counts (FPC), fluorescent bacteria were injected in the larvae and imaged using MVX10 Olympus microscope. Fluorescence was quantified by computation using Fiji (ImageJ software) as following: 1/ Background was measured in images of PBS injected larvae and then was subtracted in the fluorescence images, 2/ “make binary” function was run, and 3/ “measure area” function was used to determine the number of fluorescent pixels of the image.

### Macrophage and neutrophil ablation and overproduction

To induce macrophage depletion, 10 nl of Lipo-Clodronate or Lipo-PBS (clodronateliposomes.com) were injected intravenously (*i*.*v*.) in larvae at 1 dpf. Macrophage-depleted larvae were selected for infection based on the reduction of red-labeled macrophages *tg(mpeg1*:*mCherry-F)* 24 h after the treatment. For neutrophil depletion, 3 nl of antisense translational morpholino *csf3r* 0.7 mM (5’GAAGCACAAGCGAGACGGATGCCAT3’, Gene Tools) was microinjected in the one-cell stage *tg(mpx*:*GFP)* embryos. Standard control from Gene Tools (see Morpholino injection section) was used as a control. Neutrophils or macrophages were alternatively depleted using metronidazole treatment of *tg(mpx*:*gal4/UAS*:*nfsb-mCherry)* larvae or *tg(mpeg1*:*gal4/UAS*:*nfsb-mCherry)*, respectively (see below). Microinjection of 3 nl of 10 ng/μl of gcsf3a or gcsf3b over-expressing plasmids [60] at 1-cell stage was used to increase neutrophil supply in embryos.

### Drug treatments of zebrafish larvae and morpholino injection

For neutrophil depletion, *tg(mpx*:*Gal4/UAS*:*nfsB-mCherry)* and *tg(mpx*:*Gal4 /UAS*:*nfsB-mCherry/mpeg1*:*GFPcaax)* embryos expressing a Nitroreductase-mCherry fusion protein specifically in neutrophils, were placed in fish water containing 5 or 10 mM Metronidazole/0.1% DMSO (MTZ, Sigma-Aldrich) (freshly prepared), at 40 hpf. Treatment with 0.1% DMSO and not transgenic siblings treated with MTZ were used as controls. Higher neutrophil depletion was observed using 10 mM MTZ. Therefore, 10 mM concentration of MTZ was used for further analysis, excepted in Fig 3C where a representative larva with 50% neutrophil depletion using 5 mM MTZ is shown. For macrophage depletion, *tg(mpeg1*:*Gal4/UAS*:*nfsB-mCherry)* were treated with 10 mM Metronidazole/0.1% DMSO at 30 hpf. *tg(mpeg1*:*Gal4/UAS*:*nfsB-mCherry)* treated with 0.1% DMSO and not transgenic siblings treated with MTZ were used as controls. VAS2870 (Sigma-Aldrich SML0273) stock was prepared in DMSO at 15 mM. Two dpf *tg(lyz*:*DsRed)* embryos were injected in the yolk with 5 nl of 20 μM VAS2870 diluted in miliQ water or with 5 nl of water-diluted DMSO. Apocynin (Santa Cruz, CAS498-02-2) was dissolved at 100 mM in DMSO. *E*. *coli*-infected larvae were placed in fish water containing 250 μM Apocynin for 1 day. Decrease of superoxide production was detected using DHE (Dihydroethidium, Santa Cruz CAS104821-25-2) staining (see below). Nitric Oxide inhibition was performed with the pan-NOS inhibitor NG-Nitro-L-Arginine Methyl Ester (L-NAME) (Sigma-Aldrich, CAS 51298-62-5). After notochord infection, embryos were placed immediately in 1 mM L-NAME fish water for the whole time course of the experiments. To knock down translation of *P47*^*phox*^, the antisense oligonucleotide morpholino (5’ CGGCGAGATGAAGTGTGTGAGCGAG 3’), overlapping the AUG start codon [61] was used. 2.1 ng of *P47*^*phox*^ or Control (standard control from Gene Tools, 5' CCTCTTACCTCAGTTACAATTTATA 3') morpholinos were injected at 1-cell stage.

### Staining and immuno-labelling in whole embryo

Mpx activity and neutrophils were detected in *tg(mpx*:*GFP)/mpx+/-* and *tg(mpx*:*GFP)/mpx-/-* larvae at 1 day post *E*. *coli* injection (dpi) using Sudan black staining and anti-GFP antibody (molecular probe A11122, dilution 1/500), respectively [21]. For superoxide detection within the cells, DHE was added to the fish medium at 3 μM at 1 dpi for one hour and larvae were washed 2 times before imaging using confocal microscopy (excitation/emission 532/605 nm) [32]. To detect nitric oxide, infected *tg(lyz*:*DsRed)* embryos were stained with 4-Amino-5-methylamino-2’,7’-difluorofluorescein diacetate, Diaminofluorescein-FM diacetate (DAF-FM-DA) (Sigma, CAS 254109-22-3) [31] at 5 μM in fish medium for 2 hours at 6, 10 hpi and 1 dpi (for *E*. *coli* infection) or 2 hpi (for *Salmonella* infection). Larvae were rinsed three times in fish water before imaging using epi-fluorescence and confocal microscopy (excitation/emission: 488/515 nm). Dead cells were detected using Sytox Green staining. Larvae were injected with 3 nL of 50 μM Sytox Green (Molecular Probes) in the vein at 1 dpi and placed at 28.5°C. One hour after Sytox Green injection, larvae were mounted in 1% low-melting-point agarose and imaged using epi-fluorescence and spinning disk confocal microscopy (excitation/emission: 488/526 nm).

### Quantification of total leukocyte population, quantification of recruited neutrophils and quantification of dead cells

Tricaine-anesthetized reporter larvae were imaged using MVX10 Olympus microscope. In Figs 2, S3 and S6 total numbers of fluorescent neutrophils or macrophages were quantified as Leukocyte Units (LUs) by computation using Fiji (ImageJ software) as described in [62]. In Figs 1, 3, 5, 6, 7, 8, S2 and S9 the total number of fluorescent leukocytes were quantified by computation using Fiji (ImageJ software) as following: 1/ leukocytes were detected using “Find Maxima” function, 2/ Maxima were automatically counted using run("ROI Manager …"), roiManager("Add") and 3/ roiManager("Measure") functions. For quantification of recruited fluorescent neutrophils, tricaine-anesthetized reporter larvae were imaged using MVX10 Olympus microscope or confocal microscope. Neutrophils were directly quantified on the images, in a defined region of interest (the Notochord or muscle region as indicated in the figure diagrams). Dead cells were directly quantified on confocal images, in a defined region of interest.

### Statistics analysis

Graph Pad Prism 4.0 Software (San Diego, CA, USA) was used to construct graphs and analyze data in all figures, except Fig 6F, 6H and S9F, which were performed in Excel 2010 (Microsoft). Specific statistical tests were used to evaluate the significance of differences between groups (the test and p value are indicated in the figure legend). Outliers were determined using Grubbs' test (Graph Pad Prism 4.0 Software). The sample size is indicated in the figure legend and the sample size estimation and the power of the statistical test were computed using GPower software. Samples were allocated into experimental groups by randomization. The number of independent experiments (biological replicates) is indicated in the figure legends when applicable. The survival rate of treated embryos was compared with that of the control embryos using the log-rank (Mantel-Cox) test.

### Imaging of live zebrafish larvae

Larvae were anesthetized and mounted as previously described [21]. Epi-fluorescence microscopy was performed using a MVX10 Olympus microscope (MVPLAPO 1X objective; XC50 camera). Confocal microscopy was performed using a confocal Leica SPE upright microscope (40x HCX APO L 0.80 W and 20x CHX APO L 0.5 W objectives) and an ANDOR CSU-W1 confocal spinning disk on an inverted NIKON microscope (Ti Eclipse) with ANDOR Neo sCMOS camera (20x air/NA 0.75 objective). Image stacks for time-lapse movies were acquired at 23–26°C every 4 min, typically spanning 50 μm at 2 μm intervals, at 1024x512 or 512x512 pixel resolution. The 4D files generated from time-lapse acquisitions were processed using Image J, compressed into maximum intensity projections and cropped. Brightness, contrast, and colour levels were adjusted for maximal visibility.

### Quantitative RT-PCR analysis

For gcsf over-expression, larvae were injected with gcsf3a or gcsf3b over-expressing plasmids or no plasmid as described above. At 2 dpf, larvae were either uninfected or infected with *E*. *coli* in the notochord. To determine the relative expression of *gcsf3a*, *gcsf3b and lyz*, total RNA from infected larvae and controls (pools of 6 larvae each) was prepared at 1–2 dpi. For mpeg1 mRNA expression analysis, total RNA was extracted from 3 dpf Lipo-PBS and Lipo-clodronate treated larvae (10 larvae per pool, 3 pools per conditions). RNA preparation, reverse transcription and Q-PCR were performed as described in [63], using *ef1a* as a reference gene. Q-RT-PCR analyses were performed using LC480 software. The primers used were the following: zcsf3a.32 (5’gac tgc tct tct gat gtc tg 3’), zcsf3a.52 (5’aac tac atc tga acc tcc tg 3’), zcsf3b.31 (5’ggc agg gct cca gca gct tc 3’), zcsf3b.51 (5’gga gct ctg cgc acc caa ca 3’), LyzA (5’ccg tta cag taa gaa tcc cag g 3’) and lyzS (5’ aga att tgt gca aag tgg cc 3’), zef1a.5 (5’ ttc tgt tac ctg gca aag gg 3’), zef1a.3 (5’ ttc agt ttg tcc aac acc ca 3’), mpeg1.FW1 (5’ ttt cac ctg ctg atg ctc tg 3’) and mpeg1.RV1 (5’ atg aca tgg gtg ccg taa tc 3’).

## Supporting information

S1 Fig Comparison of *E*. *coli* K12 strain with enteric adherent invasive *E*. *coli* strains in notochord infection model. (A) Diagram showing the injection of Crimson expressing *E*. *coli* in the notochord (arrow: injection site) in triple transgenic larvae *tg(RCN3*:*gal4/UAS*:*DsRed/mpx*:*GFP)* at 2 days post-fertilization larva (dpf). (B) Larvae were analyzed by confocal microscopy at 5 hours post-injection (hpi) of either PBS or Crimson-*E*. *coli*. Notochord images are representative 3D projections of overlaid fluorescence channels: DsRed (blue), GFP (green) and Crimson (magenta). The right panel is a projection of cross-section view of the notochord in the region indicated by the dotted line. Dashed circle outlines the notochord. Scale bar: 30 μm. (C) Electron microscopy of the notochord region in infected larvae at 4 hpi. b: bacteria, nc: notochord, col: collagen sheath. Scale bar = 1μm. (D) GFP expressing Escherichia coli strains (K12, AIEC LF82, LF82-ΔlpfA and JM83-ΔmsbB) were injected in the notochord of *tg(mpx*:*GFP)* embryos at 2 dpf. GFP (*E*. *coli* and neutrophils) was analysed by fluorescence microscopy at 1 dpi. In AIEC LF82, LF82-ΔlpfA and JM83-ΔmsbB infections, bacteria were cleared and neutrophil recruitment to the notochord (N) was induced similarly to K12 infections (arrowheads). Scale bar: 400 μm. (E) Survival curves of zebrafish larvae that have been infected in their notochord with indicated *Escherichia coli* strains from 0 to 2 dpi (Log rank test, ns = not significant p>0.05, *N* is indicated on in the figure).
(PDF)

S2 Fig Macrophages ablation using Nitroreductase/Metronidazole system does not affect bacterial growth during notochord infection. (A-B) *Tg(mpeg1*:*Gal4/UAS*:*nfsB-mCherry)* larvae were treated either with DMSO or Metronidazole (MTZ) added in fish water at 35 hpf. Treated larvae were imaged at 0, 1 and 2 days post-treatment (dpT) using fluorescence microscopy. (A) Quantification of total macrophages in DMSO and MTZ treated larvae at 0 and 1 and 2 dpT (Mean number of cell/larva ± SEM, *N*_*DMSO*_ *=* 5 and *N*_*MTZ*_ *=* 5, three independent experiments, Mann-Whitney test, one-tailed, **p<0.*005*). (B) Representative fluorescent images (DsRed) of DMSO and MTZ treated larvae at 1 dpT. Asterisk: auto-fluorescence of the yolk. Scale bar: 600 μm. (C) At 1 dpT larvae were infected with *E*. *Coli*-GFP in the notochord. Representative fluorescent images (GFP) showing infection outcome at 0 and 1 dpi for two indicated conditions. Asterisk: auto-fluorescence of the yolk, white arrowhead: *E*. *Coli*-GFP injection site. Scale bar: 600 μm. (D) Bacterial load quantification by Fluorescent Pixel Count (FPC) in MTZ treated *Tg(mpeg1*:*Gal4/UAS*:*nfsB-mCherry)* (nfsB^+^ MTZ) at 1 dpi showing no significant differences in the bacterial load with control groups (*Tg(mpeg1*:*Gal4/UAS*:*nfsB-mCherry)* treated with DMSO referred as nfsB^+^ DMSO and non transgenic siblings treated with MTZ referred as nfsB^-^ MTZ) (mean values ± SEM, Kruskall-Wallis test with Dunn’s post-test, *N*_*nfsB+ DMSO*_ = 13, *N*_*nfsB- MTZ*_ *=* 7, *N*_*nfsB+ MTZ*_ *=* 13).
(PDF)

S3 Fig Embryos need correct neutrophil density to fight notochord infection. Two dpf *tg(mpx*:*GFP)* embryos were infected in the notochord with low dose (< 3000 CFUs) (A, B), high dose (> 4000 CFUs) (C) or very high dose (>7000 CFUs) (D). (B) To decrease neutrophil density, *tg(mpx*:*GFP)* embryos were injected at the one cell stage with the *csf3r* morpholino and then infected in the notochord at 2 dpf with a low dose of red fluorescent *E*. *coli*-DsRed. (D) To increase neutrophil density, *tg(mpx*:*GFP)* embryos were injected at the one cell stage with the *gcsfa* over-expressing plasmid and then infected in the notochord at 2 dpf with a very high dose of red fluorescent *E*. *coli*-DsRed. Charts show the quantification of CFU (red-bar charts) and of the total neutrophil number (green-bar charts) at 0, 1 and 2 dpi (Mann-Whitney test, two-tailed, *N*_*larvae*_ is indicated on the columns, *p<0.05, ** p<0.01 and ***p<0.001). Larvae images are representative overlays of fluorescence (green: neutrophils and red: *E*. *coli*) and transmitted light images at 2 dpi (asterisk: auto-fluorescence of the yolk).
(PDF)

S4 Fig *gcsfa*, *gcsfb* and *lyz* expressions upon injection of gcsfa and gcsfb plasmids in zebrafish embryos and effects of gcsfb overexpression during notochord infection. qRT-PCR of *gcsfa* (A), *gcsfb* (B) and *lyz* (C) mRNAs relative to *ef1a* in wild type larvae or in larvae expressing a *gcsfa-* or *gcsfb-* transgenes. Embryos were either uninjected (CTRL) or injected with a *gcsfa*- or *gcsfb-*overexpressing plasmid at one cell-stage. They were subsequently either uninfected or infected with *E*. *coli* in the notochord at 2 dpf. RNA was extracted from whole larvae at 1–2 dpi (6 larvae per pool, mean ± SEM, *N =* 2–4). (D-F) Two dpf *tg(mpx*:*GFP)* embryos overexpressing *gcsfb* were either uninjected or infected in the notochord with a high dose of fluorescent *E*. *coli*-DsRed (>4000 CFU). (D) Larvae images are representative overlays of fluorescence (*E*. *coli*) and transmitted light images at 2 hpi and 2 dpi, showing the disappearance of bacteria at 2 dpi. Arrowhead shows the injection site. Scale bars = 400μm (N_larvae_ = 6). (E-F) Quantification of neutrophil population at 2, 3 and 4 dpf in uninfected larvae (E) and at 0, 1 and 2 dpi in *E*. *coli* infected larvae (F) (Mann-Whitney test, two-tailed, *N*_*larvae*_ is indicated on the columns, ***p<0.*001*).
(PDF)

S5 Fig Notochord infection induces neutrophils death. *Tg(lyz*:*DsRed)* larvae were either injected with PBS (A) or infected with low dose (LD) (B) or high dose (HD) (C) of *E*. *coli* in the notochord. Neutrophils were detected using DsRed (red) and dead cells using Sytox Green (green) at 24 hpi and trunk regions were imaged using Spinning Disk Confocal microscopy. Representative maximal projections of confocal montages show increased cell death, including dead neutrophils around the notochord in HD infection, comparing to LD and PBS injection. White stars show non-specific staining in the yolk extension and neurones of the spinal cord. Arrowheads show Sytox Green injection sites. White boxes in the left panels show the zoomed areas (right panels). Scale bars: 50 μm for the left panels and 25 μm for the right panels. (D) Number of Sytox Green positive cells and (E) Sytox Green positive neutrophils around the notochord in indicated conditions (mean number of cell/larva ± SEM, *N*_*PBS*_ = 9, *N*_*LD*_ = 9 and *N*_*HD*_ = 8, from two independent experiments, Kruskal Wallis test with Dunn’s post-test, *p<0.*05*, ***p<0.*001*).
(PDF)

S6 Fig High dose infection in the notochord leads to increased macrophage population. (A) Two dpf *tg(mpeg1*:*mCherry-F)* larvae were injected in the notochord either with low dose (LD) or high dose (HD) *E*. *coli-GFP*. Trunk regions were imaged using fluorescence microscopy at 1 dpi. Scale bar: 200 μM. Representative fluorescence (mCherry and GFP) overlaid with bright field images shows macrophage accumulation around the notochord in both LD and HD infections. Bacteria proliferate in HD infection. (B) Two dpf *tg(mfap4*:*mCherry-F)* larvae were injected in the notochord either with PBS or high dose *E*. *coli-GFP*. Counts of macrophages in the trunk and tail region by Leukocyte Unite quantification (LU) in indicated conditions (mean values ± SEM, Mann Whitney’s test, two-tailed, *N*_*PBS*_ = 12–14 and *N*_*HD*_ = 6–9, *p<0.*05*). Larva diagram shows the region of counting.
(PDF)

S7 Fig Recruited neutrophils do not produce detectable Nitric Oxide. (A) Nitric oxide is produced by neutrophils in the AGM following *Salmonella Typhimurium* infection. Two dpf *tg(lyz*:*DsRed)* embryos were infected in the hindbrain or in the notochord with *Salmonella Typhimurium*. At 2 dpi Nitric oxide was detected with DAF-FM-DA (green) using confocal microscopy. Representative overlay of maximum projections of multi scan acquisitions (DsRed and DAF-FM-DA) with transmitted light images shows that Nitric oxide is produced by neutrophils in the AGM (A left panel) and in the notochord (A right panel), but not in the recruited neutrophils (A right panel) (*N*_*hindbrain*_ *= 3* And *N*_*notochord*_ *= 3*). (B) Two dpf *tg(lyz*:*DsRed)* embryos were infected with *E*. *coli*-GFP in the notochord. (B1, B2) Representative overlay of maximum projections of multi-scan acquisitions (DsRed and DAF-FM-DA) with transmitted light images shows that Nitric oxide (green) is produced constitutively in the notochord (white arrowheads) but not in recruited neutrophils at 6 hpi (pink arrowheads). (B3-B6) Trunk images are representative DAF-FM-DA fluorescence (B3-B4) and DsRed fluorescence images (B5-B6) from PBS- or *E*. *coli-*injected embryos at 1 dpi. *N*_*PBS*_ *= 2* and *N*_*E*.*coli*_ *= 10*, AGM: Aorta-gonad-mesonephros, NC: notochord, scale bars: 30 μm. (C) Two dpf *tg(lyz*:*DsRed)* embryos were infected in the notochord with *E*. *coli-*GFP and then immediately treated with either L-NAME or water (CTRL). Bacteria in the whole larvae were imaged using fluorescent microscopy at 0 and 1 dpi and bacterial burden were quantified by Fluorescent Pixel Count (FPC) (horizontal lines indicate the median values, *N*_*CTRL*_ = 9–11 and *N*_*L-NAME*_ = 10–11, representative of 4 independent experiments, Kruskal-Wallis’ test with Dunn’s post-test, ns: not significant, *p>0*.*05*).
(PDF)

S8 Fig NADPH oxidase inhibitor Apocynin does not affect muscle infection outcome. (A-B-C) *E*. *coli-*DsRed were injected in the muscle of 2 dpf *tg(mpx*:*GFP)* embryos in DMSO or Apocynin treatment conditions. Bacteria (red) in the trunk region were imaged using fluorescent microscopy at 0 dpi and 1 dpi. (A) Representative bright field images overlaid with fluorescent channel of DMSO and Apocynin treated larvae. (B) Quantification of bacterial burden by Fluorescent Pixel Count (FPC) in indicated conditions (horizontal lines indicate the median values, *N*_*DMSO*_ = 18–19 and *N*_*APO*_ = 16–17, Kruskal-Wallis’ test with Dunn’s post-test, *** *p<0*.*001*). Larva diagram shows the region of counting. (C) Survival curves of DMSO and Apocynin treated larvae infected with *E*. *coli* in the muscle from 0 to 3 dpi or injected with PBS (*N*_*larvae*_ is indicated in the figure, log rank test, *p>0*.*05*, ns: not significant).
(PDF)

S9 Fig NADPH oxidase inhibitor VAS2870 increases the susceptibility of larvae to notochord infection. (A) Experimental scheme. VAS2870 or DMSO was injected in the yolk of *tg(lyz*:*DsRed)* or *tg(mpx*:*GFP)* embryos at 2 dpf. One hour later, fluorescent *E*. *coli* bacteria were injected in the notochord and the injected embryos were scored from 1 dpi. (B) Representative fluorescent images of neutrophils in the VAS2870 or DMSO treated *tg(mpx*:*GFP)* embryos at 1 day post treatment (dpT) without bacterial injections. Scale bar: 400 μm. (C) Counts of total neutrophil population in indicated conditions at 6 hours (hpT) and 1 dpT (mean ± SEM, *N*_*DMSO*_ = 29–30 and *N*_*VAS2870*_ = 25–29, Mann-Whitney test, two-tailed, *p>0*.*05*, *ns = not significant*). (D) Trunk images are representative overlays of DsRed (neutrophils), GFP (*E*. *coli*) and transmitted light images at 1 dpi in PBS- or *E*. *coli*- injected larvae in DMSO or VAS2870 treatment conditions. Scale bars: 100 μm. White arrowheads: *E*. *coli* in the notochord. (E) Survival curves of larvae injected with either PBS or *E*. *coli* from 0 to 3 dpi in DMSO or VAS2870 treatments (*N*_*larvae*_ is indicated in the figure, log rank test, ***p<0.*001*, from three independent experiments). (F) Larva phenotypes and bacterial outcome were scored from 0 to 3 dpi. (w/o: without bacterial growth, the number of larvae (*N*) is indicated each the column, from three independent experiments).
(PDF)

S10 Fig *p47*^*phox*^ morpholino deceases superoxide production in activated neutrophils. *Tg(mpx*:*GFP)* embryos were injected at the one cell stage with either *p47*^*phox*^ morpholino (MO *p47*^*phox*^) or a control morpholino (MO CTRL). Morphants were infected in the muscle with GFP- *E*. *coli* and superoxide was detected with DHE at 3 hpi. (A) Larvae images are representative fluorescence images of DHE at 3 hpi. Scale bar: 100 μm. White boxes indicate the position of the confocal images in (B). (B) Representative overlay of GFP fluorescence (neutrophils+E. coli) with DHE fluorescence (maximal projections of confocal images) show superoxide in neutrophils in control morphants but not in *p47*^*phox*^ morphants. Scale bars: 50 μm. (C-D) Dot plots are quantification of recruited DHE^+^ cells (C), recruited neutrophils (D), and recruited DHE^+^ MPX^+^ cells (E) in CTRL and *p47*^*phox*^ morphants (mean number of cell/larva ± SEM, *N*_*MO CTRL*_ = 13, *N*_*MO P47*_ = 13, Mann-Whitney test, two-tailed, ***p<0.*001*, ns: *p>0*.*05* non significant). Diagrams show the counting region.
(PDF)

S1 Video Neutrophils produce superoxide in phagosomes containing *E*. *coli*. Two dpf transgenic embryos *tg(mpx*:*GFP)* were injected in the notochord with *E*. *coli* expressing green fluorescent protein. Due to the high pressure of the injection and the resistance of the notochord, small amount of bacteria is dispersed at the injection site and phagocytosed by neutrophils (green). Superoxide was detected using Dihydroethidium (DHE, red). Representative time-lapse started at 6 hpi during 16 mins. Image stacks were acquired every 4 minutes at 2 μm interval at 1024x256 pixel resolution using confocal Leica SPE upright microscope with 40x HCX APO L 0.80 W objective. White arrows show superoxide in phagosomes bearing bacteria in recruited neutrophils at the injection site. Time code in minute. Scale bar: 15 μm.
(AVI)
