# Supplementary figures and images for: Neutrophils use superoxide to control bacterial infection at a distance
Source: PLoS Pathog. 2018 Jul 17;14(7):e1007157. doi: 10.1371/journal.ppat.1007157 (PMC6049935; doi:10.1371/journal.ppat.1007157)

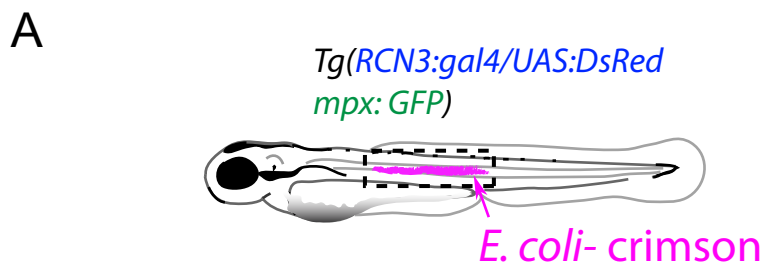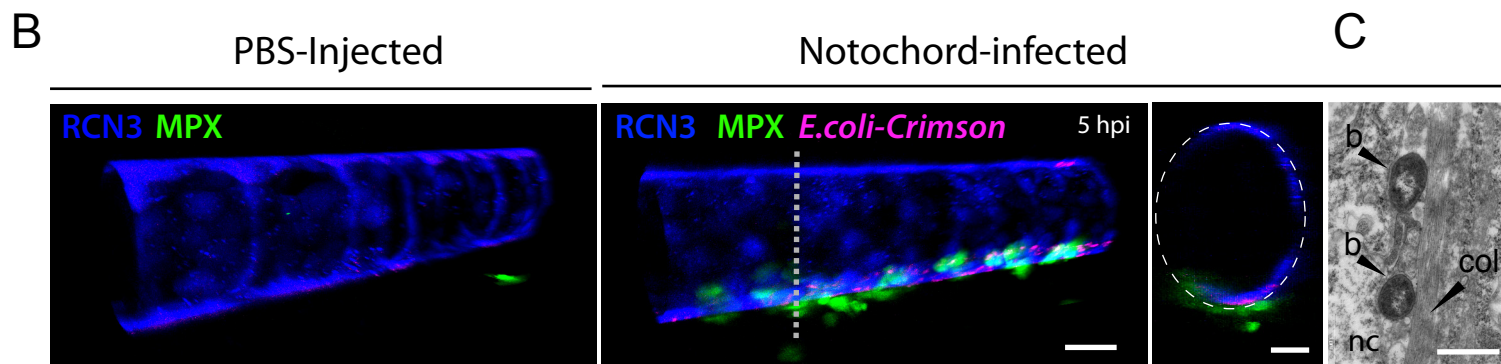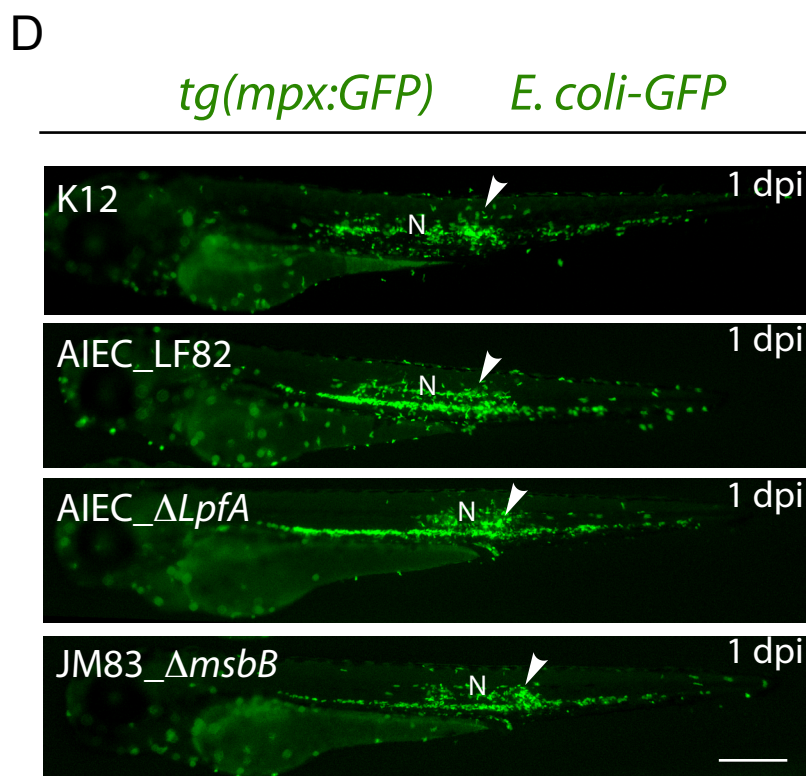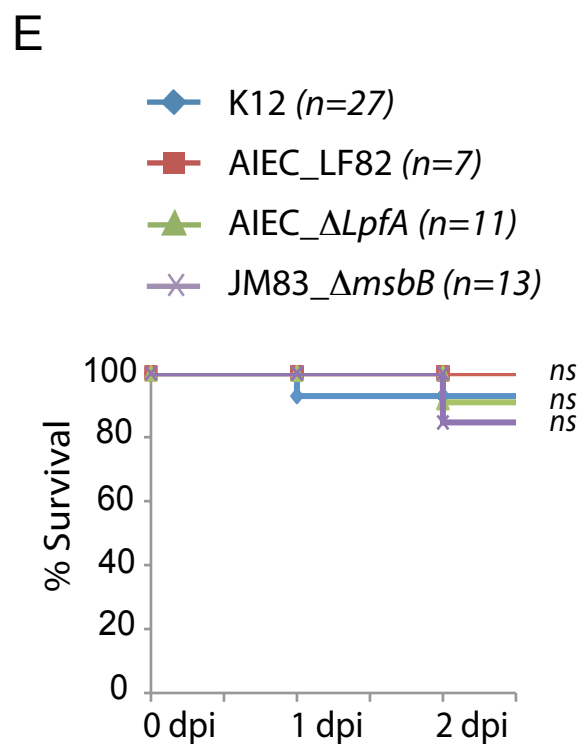

Supplement: S1 Fig — (A) Diagram showing the injection of Crimson expressing E. coli in the notochord (arrow: injection site) in triple transgenic larvae tg(RCN3:gal4/UAS:DsRed/mpx:GFP) at 2 days post-fertilization larva (dpf). (B) Larvae were analyzed by confocal microscopy at 5 hours post-injection (hpi) of either PBS or Crimson-E. coli. Notochord images are representative 3D projections of overlaid fluorescence channels: DsRed (blue), GFP (green) and Crimson (magenta). The right panel is a projection of cross-section view of the notochord in the region indicated by the dotted line. Dashed circle outlines the notochord. Scale bar: 30 μm. (C) Electron microscopy of the notochord region in infected larvae at 4 hpi. b: bacteria, nc: notochord, col: collagen sheath. Scale bar = 1μm. (D) GFP expressing Escherichia coli strains (K12, AIEC LF82, LF82-ΔlpfA and JM83-ΔmsbB) were injected in the notochord of tg(mpx:GFP) embryos at 2 dpf. GFP (E. coli and neutrophils) was analysed by fluorescence microscopy at 1 dpi. In AIEC LF82, LF82-ΔlpfA and JM83-ΔmsbB infections, bacteria were cleared and neutrophil recruitment to the notochord (N) was induced similarly to K12 infections (arrowheads). Scale bar: 400 μm. (E) Survival curves of zebrafish larvae that have been infected in their notochord with indicated Escherichia coli strains from 0 to 2 dpi (Log rank test, ns = not significant p>0.05, N is indicated on in the figure). (PDF) [file ppat.1007157.s001.pdf]

A

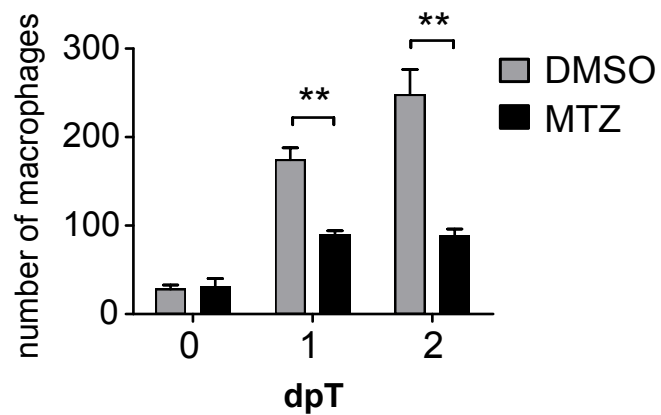

B

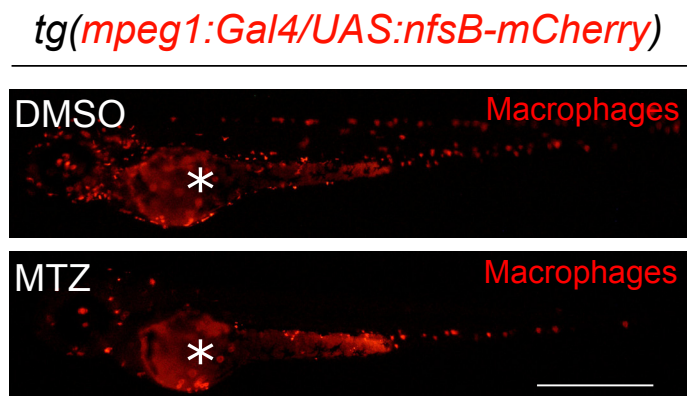

C

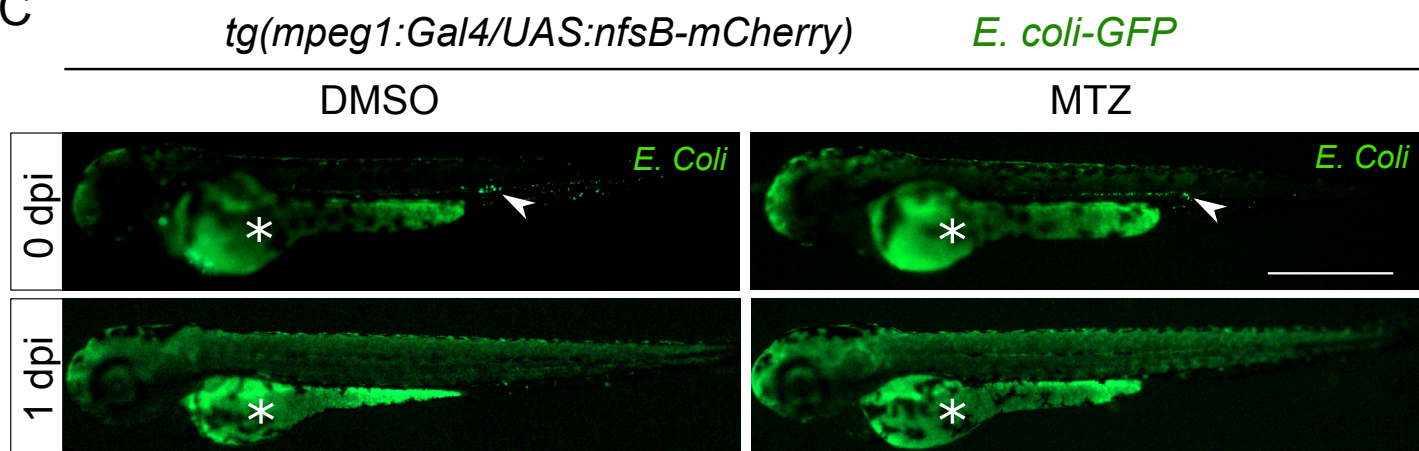

D

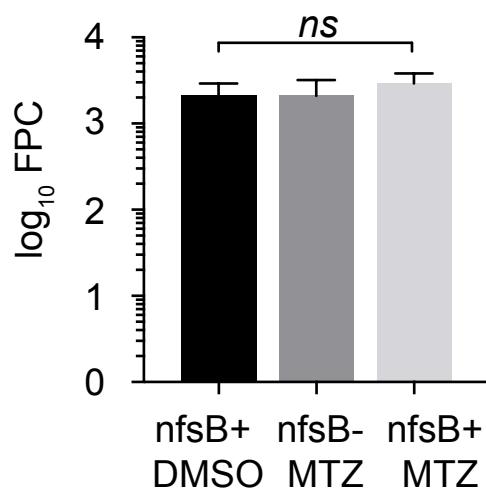

Supplement: S2 Fig — (A-B) Tg(mpeg1:Gal4/UAS:nfsB-mCherry) larvae were treated either with DMSO or Metronidazole (MTZ) added in fish water at 35 hpf. Treated larvae were imaged at 0, 1 and 2 days post-treatment (dpT) using fluorescence microscopy. (A) Quantification of total macrophages in DMSO and MTZ treated larvae at 0 and 1 and 2 dpT (Mean number of cell/larva ± SEM, NDMSO = 5 and NMTZ = 5, three independent experiments, Mann-Whitney test, one-tailed, **p<0.005). (B) Representative fluorescent images (DsRed) of DMSO and MTZ treated larvae at 1 dpT. Asterisk: auto-fluorescence of the yolk. Scale bar: 600 μm. (C) At 1 dpT larvae were infected with E. Coli-GFP in the notochord. Representative fluorescent images (GFP) showing infection outcome at 0 and 1 dpi for two indicated conditions. Asterisk: auto-fluorescence of the yolk, white arrowhead: E. Coli-GFP injection site. Scale bar: 600 μm. (D) Bacterial load quantification by Fluorescent Pixel Count (FPC) in MTZ treated Tg(mpeg1:Gal4/UAS:nfsB-mCherry) (nfsB+ MTZ) at 1 dpi showing no significant differences in the bacterial load with control groups (Tg(mpeg1:Gal4/UAS:nfsB-mCherry) treated with DMSO referred as nfsB+ DMSO and non transgenic siblings treated with MTZ referred as nfsB- MTZ) (mean values ± SEM, Kruskall-Wallis test with Dunn’s post-test, NnfsB+ DMSO = 13, NnfsB- MTZ = 7, NnfsB+ MTZ = 13). (PDF) [file ppat.1007157.s002.pdf]

A

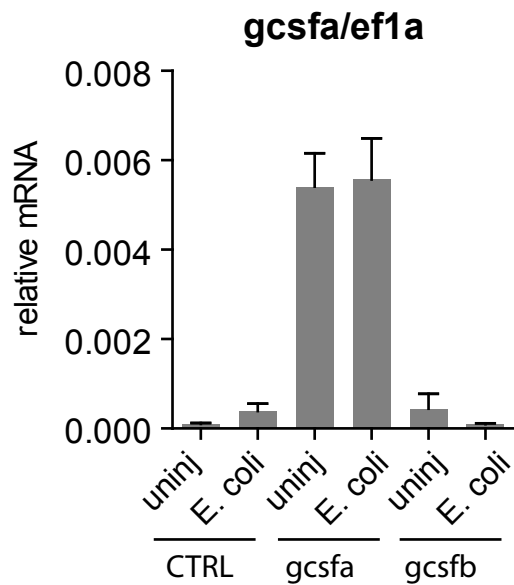

B

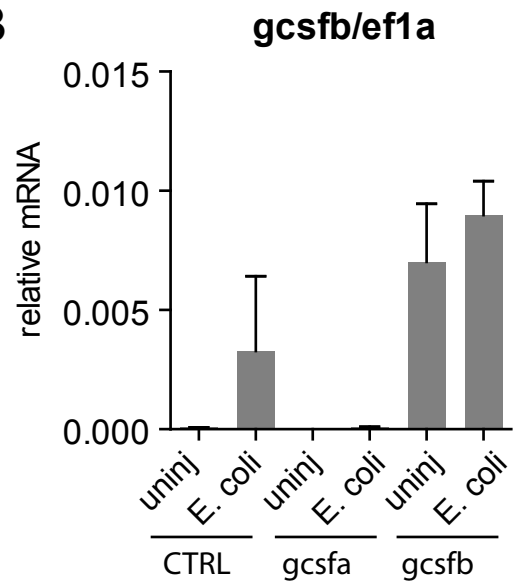

C

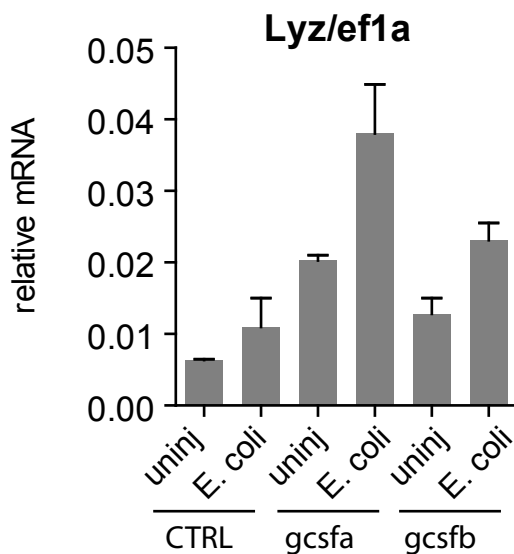

D

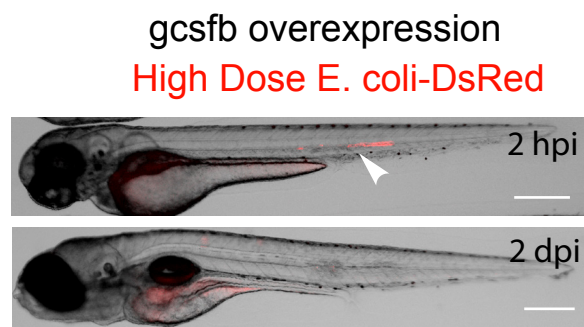

E

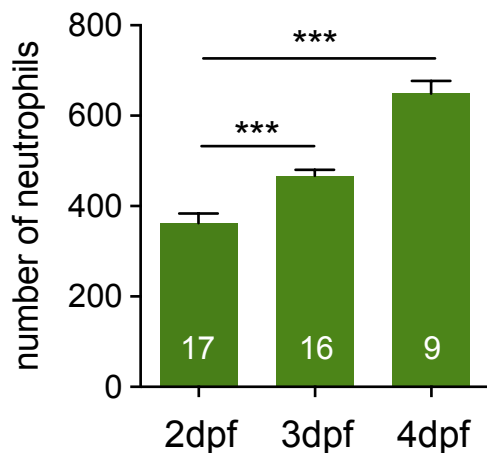

F

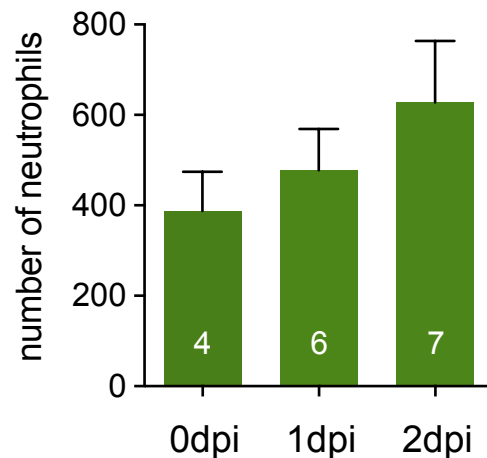

Supplement: S4 Fig — qRT-PCR of gcsfa (A), gcsfb (B) and lyz (C) mRNAs relative to ef1a in wild type larvae or in larvae expressing a gcsfa- or gcsfb- transgenes. Embryos were either uninjected (CTRL) or injected with a gcsfa- or gcsfb-overexpressing plasmid at one cell-stage. They were subsequently either uninfected or infected with E. coli in the notochord at 2 dpf. RNA was extracted from whole larvae at 1–2 dpi (6 larvae per pool, mean ± SEM, N = 2–4). (D-F) Two dpf tg(mpx:GFP) embryos overexpressing gcsfb were either uninjected or infected in the notochord with a high dose of fluorescent E. coli-DsRed (>4000 CFU). (D) Larvae images are representative overlays of fluorescence (E. coli) and transmitted light images at 2 hpi and 2 dpi, showing the disappearance of bacteria at 2 dpi. Arrowhead shows the injection site. Scale bars = 400μm (Nlarvae = 6). (E-F) Quantification of neutrophil population at 2, 3 and 4 dpf in uninfected larvae (E) and at 0, 1 and 2 dpi in E. coli infected larvae (F) (Mann-Whitney test, two-tailed, Nlarvae is indicated on the columns, ***p<0.001). (PDF) [file ppat.1007157.s004.pdf]

*tg(lyz:DsRed)* Sytox Green

24hpi

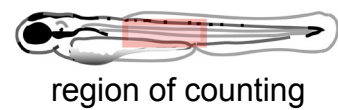

A

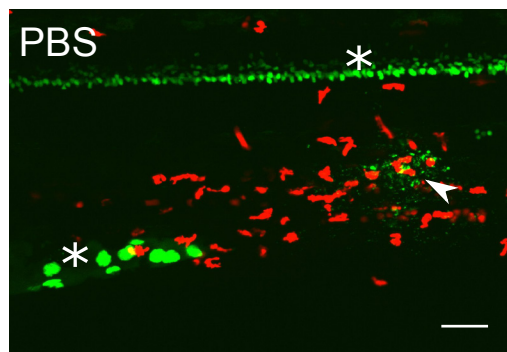

B

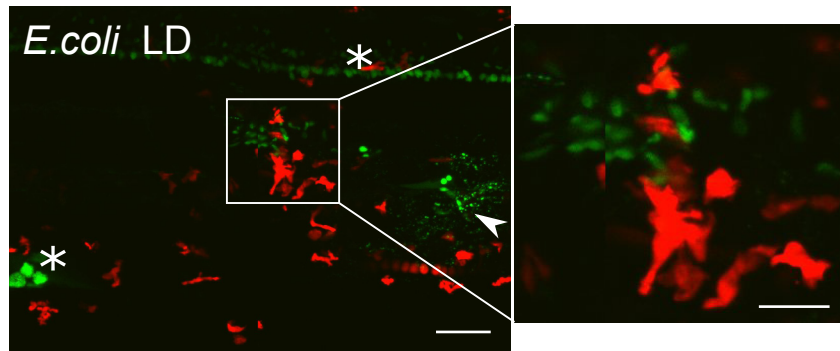

C

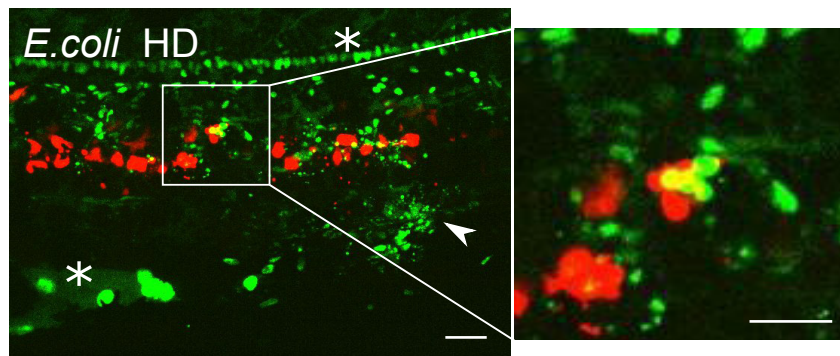

D

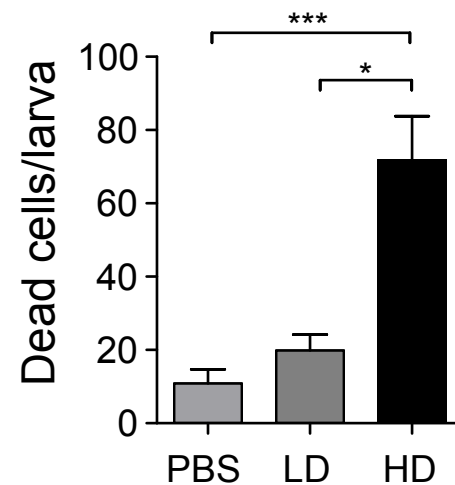

E

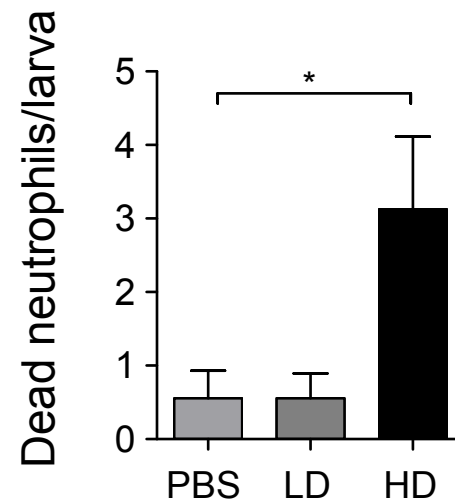

Supplement: S5 Fig — Tg(lyz:DsRed) larvae were either injected with PBS (A) or infected with low dose (LD) (B) or high dose (HD) (C) of E. coli in the notochord. Neutrophils were detected using DsRed (red) and dead cells using Sytox Green (green) at 24 hpi and trunk regions were imaged using Spinning Disk Confocal microscopy. Representative maximal projections of confocal montages show increased cell death, including dead neutrophils around the notochord in HD infection, comparing to LD and PBS injection. White stars show non-specific staining in the yolk extension and neurones of the spinal cord. Arrowheads show Sytox Green injection sites. White boxes in the left panels show the zoomed areas (right panels). Scale bars: 50 μm for the left panels and 25 μm for the right panels. (D) Number of Sytox Green positive cells and (E) Sytox Green positive neutrophils around the notochord in indicated conditions (mean number of cell/larva ± SEM, NPBS = 9, NLD = 9 and NHD = 8, from two independent experiments, Kruskal Wallis test with Dunn’s post-test, *p<0.05, ***p<0.001). (PDF) [file ppat.1007157.s005.pdf]

A

*tg(mpeg1:mCherry-F)* *E. coli-GFP*

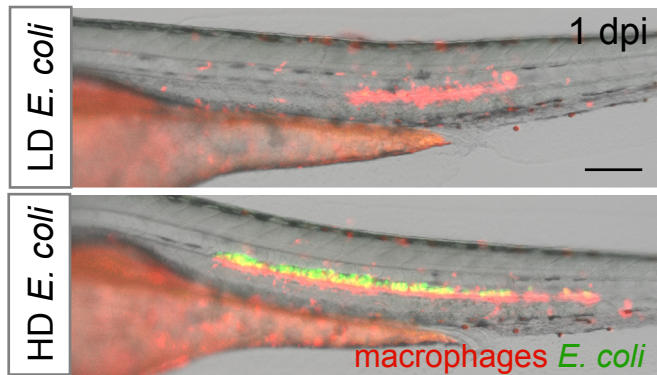

B

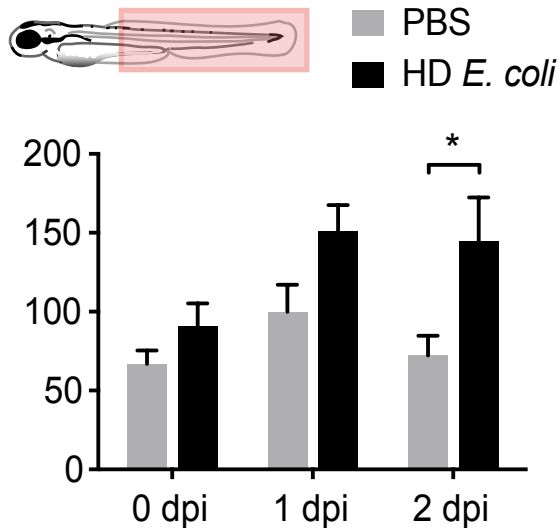

Supplement: S6 Fig — (A) Two dpf tg(mpeg1:mCherry-F) larvae were injected in the notochord either with low dose (LD) or high dose (HD) E. coli-GFP. Trunk regions were imaged using fluorescence microscopy at 1 dpi. Scale bar: 200 μM. Representative fluorescence (mCherry and GFP) overlaid with bright field images shows macrophage accumulation around the notochord in both LD and HD infections. Bacteria proliferate in HD infection. (B) Two dpf tg(mfap4:mCherry-F) larvae were injected in the notochord either with PBS or high dose E. coli-GFP. Counts of macrophages in the trunk and tail region by Leukocyte Unite quantification (LU) in indicated conditions (mean values ± SEM, Mann Whitney’s test, two-tailed, NPBS = 12–14 and NHD = 6–9, *p<0.05). Larva diagram shows the region of counting. (PDF) [file ppat.1007157.s006.pdf]

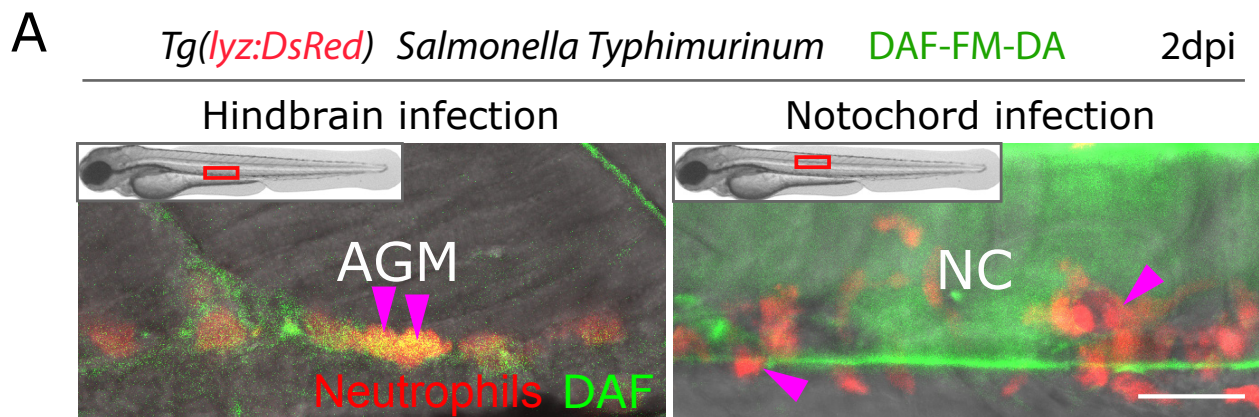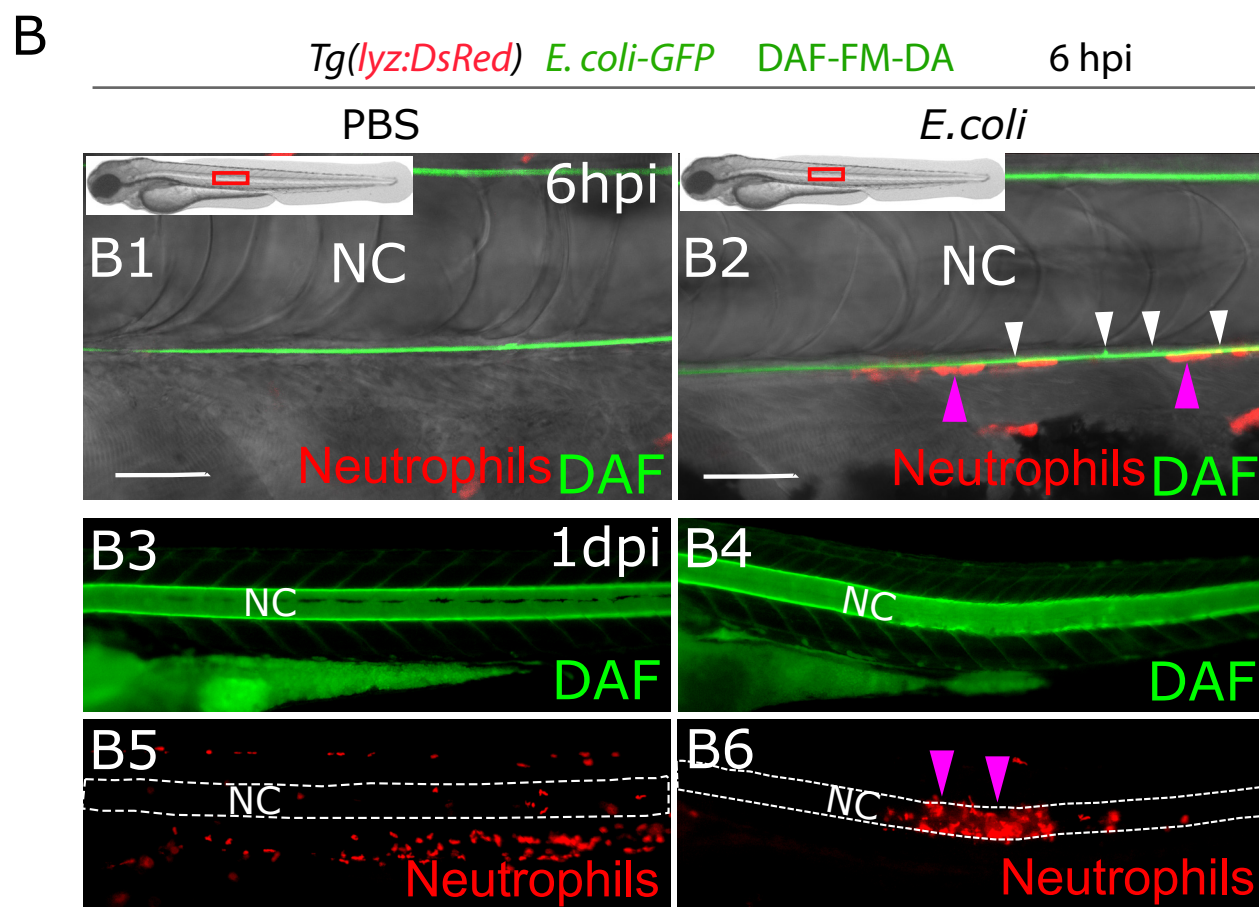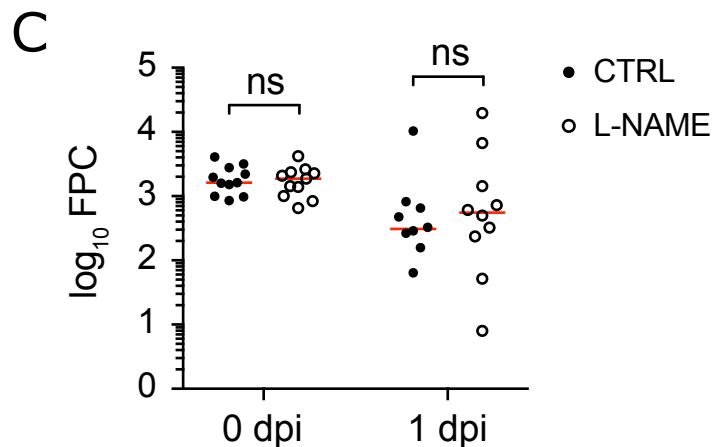

Supplement: S7 Fig — (A) Nitric oxide is produced by neutrophils in the AGM following Salmonella Typhimurium infection. Two dpf tg(lyz:DsRed) embryos were infected in the hindbrain or in the notochord with Salmonella Typhimurium. At 2 dpi Nitric oxide was detected with DAF-FM-DA (green) using confocal microscopy. Representative overlay of maximum projections of multi scan acquisitions (DsRed and DAF-FM-DA) with transmitted light images shows that Nitric oxide is produced by neutrophils in the AGM (A left panel) and in the notochord (A right panel), but not in the recruited neutrophils (A right panel) (Nhindbrain = 3 And Nnotochord = 3). (B) Two dpf tg(lyz:DsRed) embryos were infected with E. coli-GFP in the notochord. (B1, B2) Representative overlay of maximum projections of multi-scan acquisitions (DsRed and DAF-FM-DA) with transmitted light images shows that Nitric oxide (green) is produced constitutively in the notochord (white arrowheads) but not in recruited neutrophils at 6 hpi (pink arrowheads). (B3-B6) Trunk images are representative DAF-FM-DA fluorescence (B3-B4) and DsRed fluorescence images (B5-B6) from PBS- or E. coli-injected embryos at 1 dpi. NPBS = 2 and NE.coli = 10, AGM: Aorta-gonad-mesonephros, NC: notochord, scale bars: 30 μm. (C) Two dpf tg(lyz:DsRed) embryos were infected in the notochord with E. coli-GFP and then immediately treated with either L-NAME or water (CTRL). Bacteria in the whole larvae were imaged using fluorescent microscopy at 0 and 1 dpi and bacterial burden were quantified by Fluorescent Pixel Count (FPC) (horizontal lines indicate the median values, NCTRL = 9–11 and NL-NAME = 10–11, representative of 4 independent experiments, Kruskal-Wallis’ test with Dunn’s post-test, ns: not significant, p>0.05). (PDF) [file ppat.1007157.s007.pdf]

A

*E.coli*-DsRed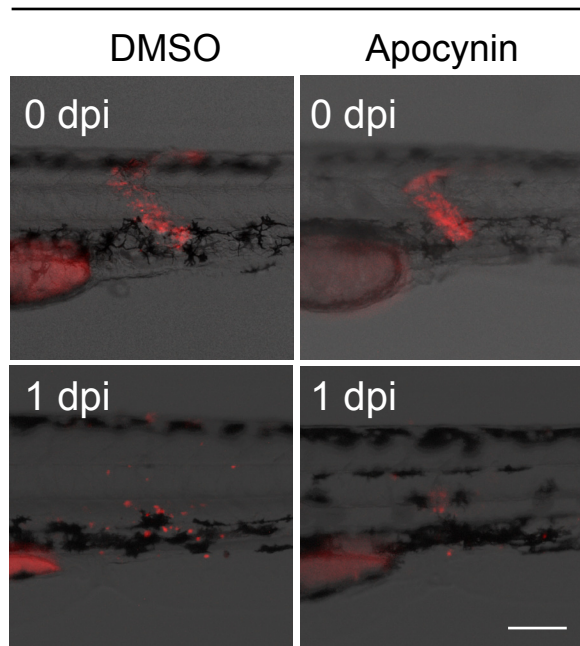

B

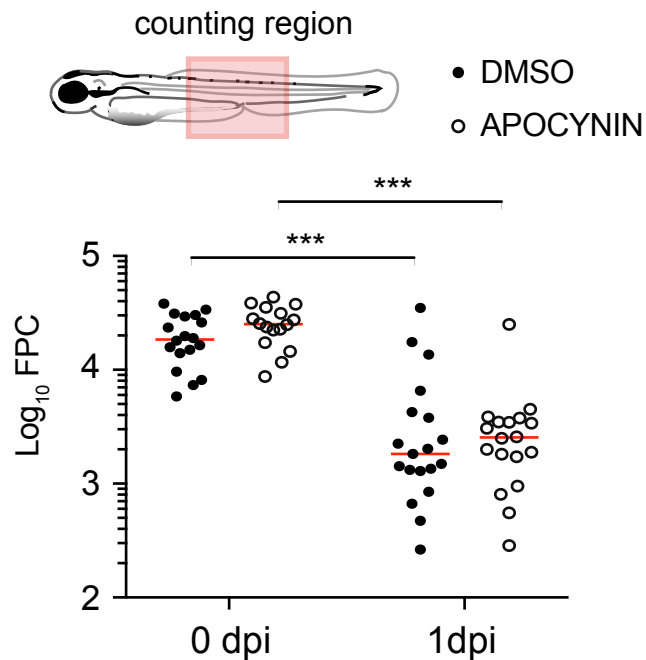

C

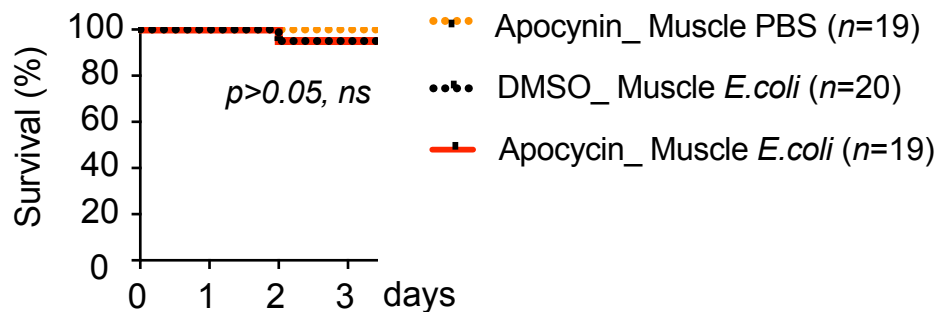

Supplement: S8 Fig — (A-B-C) E. coli-DsRed were injected in the muscle of 2 dpf tg(mpx:GFP) embryos in DMSO or Apocynin treatment conditions. Bacteria (red) in the trunk region were imaged using fluorescent microscopy at 0 dpi and 1 dpi. (A) Representative bright field images overlaid with fluorescent channel of DMSO and Apocynin treated larvae. (B) Quantification of bacterial burden by Fluorescent Pixel Count (FPC) in indicated conditions (horizontal lines indicate the median values, NDMSO = 18–19 and NAPO = 16–17, Kruskal-Wallis’ test with Dunn’s post-test, *** p<0.001). Larva diagram shows the region of counting. (C) Survival curves of DMSO and Apocynin treated larvae infected with E. coli in the muscle from 0 to 3 dpi or injected with PBS (Nlarvae is indicated in the figure, log rank test, p>0.05, ns: not significant). (PDF) [file ppat.1007157.s008.pdf]

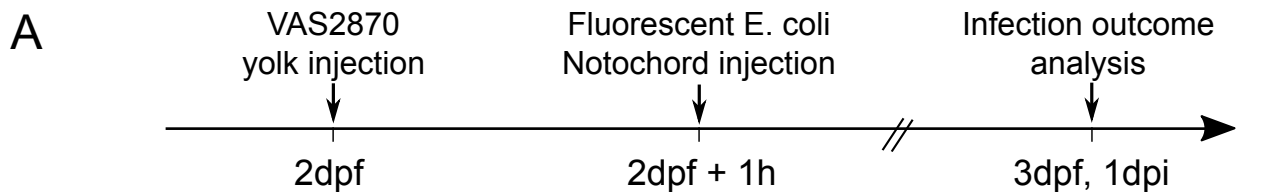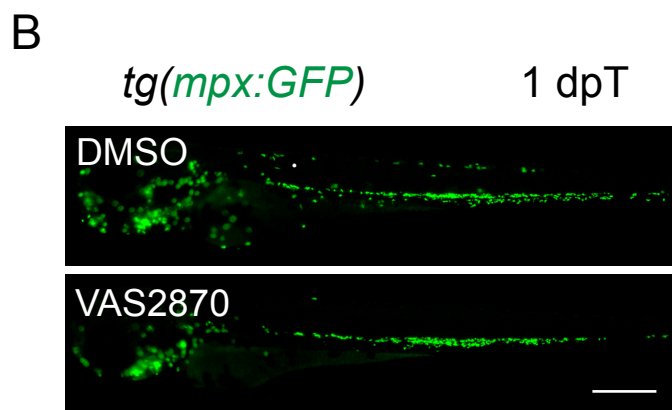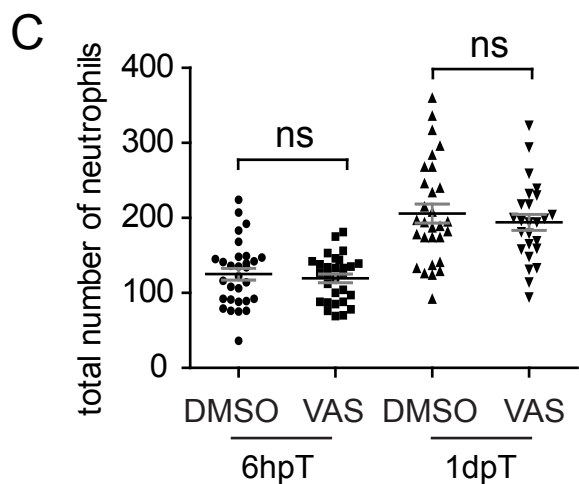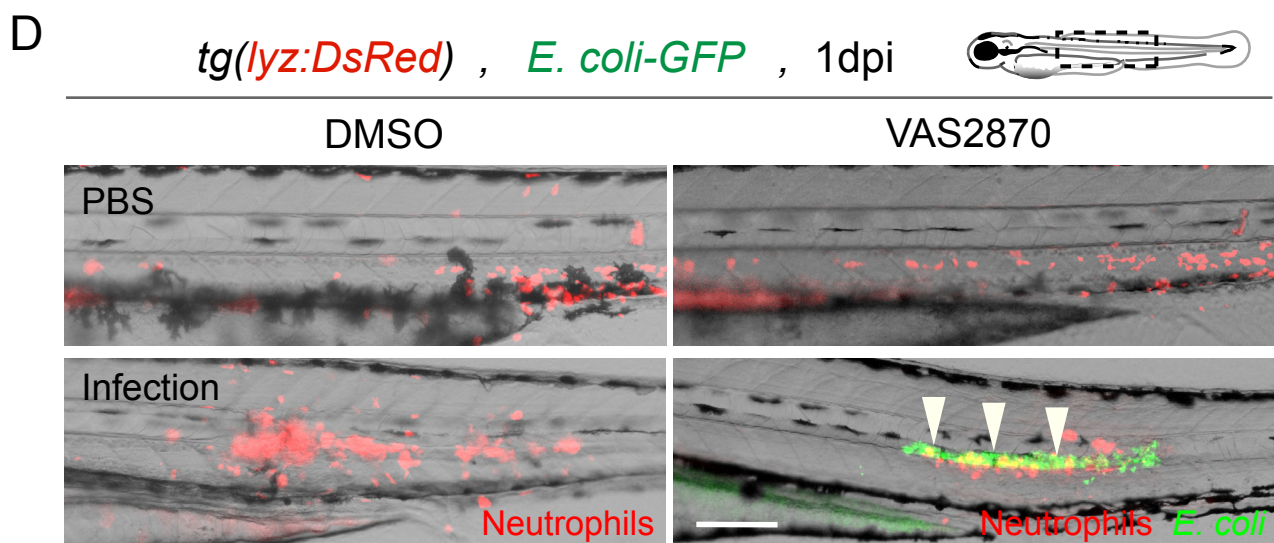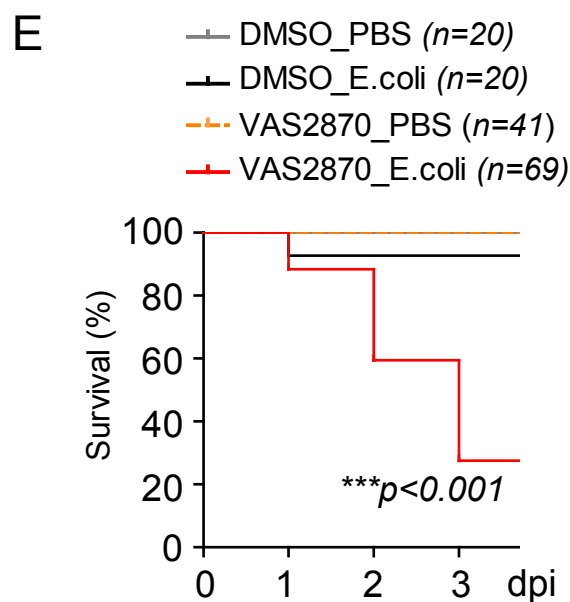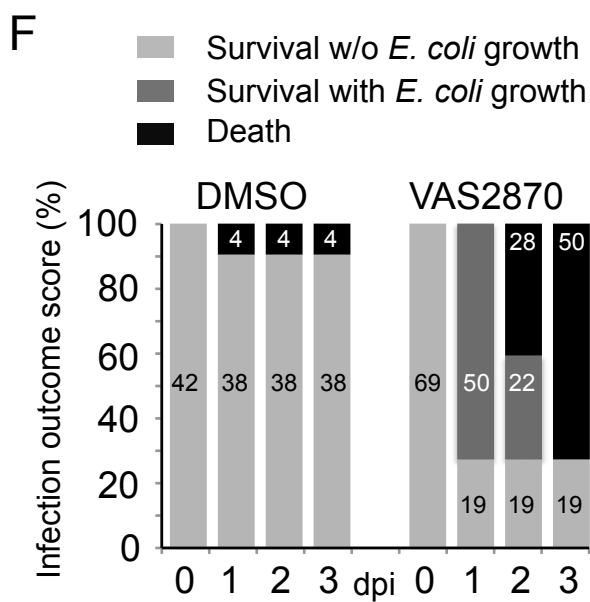

Supplement: S9 Fig — (A) Experimental scheme. VAS2870 or DMSO was injected in the yolk of tg(lyz:DsRed) or tg(mpx:GFP) embryos at 2 dpf. One hour later, fluorescent E. coli bacteria were injected in the notochord and the injected embryos were scored from 1 dpi. (B) Representative fluorescent images of neutrophils in the VAS2870 or DMSO treated tg(mpx:GFP) embryos at 1 day post treatment (dpT) without bacterial injections. Scale bar: 400 μm. (C) Counts of total neutrophil population in indicated conditions at 6 hours (hpT) and 1 dpT (mean ± SEM, NDMSO = 29–30 and NVAS2870 = 25–29, Mann-Whitney test, two-tailed, p>0.05, ns = not significant). (D) Trunk images are representative overlays of DsRed (neutrophils), GFP (E. coli) and transmitted light images at 1 dpi in PBS- or E. coli- injected larvae in DMSO or VAS2870 treatment conditions. Scale bars: 100 μm. White arrowheads: E. coli in the notochord. (E) Survival curves of larvae injected with either PBS or E. coli from 0 to 3 dpi in DMSO or VAS2870 treatments (Nlarvae is indicated in the figure, log rank test, ***p<0.001, from three independent experiments). (F) Larva phenotypes and bacterial outcome were scored from 0 to 3 dpi. (w/o: without bacterial growth, the number of larvae (N) is indicated each the column, from three independent experiments). (PDF) [file ppat.1007157.s009.pdf]

A

*Tg(mpx:GFP) - E. coli-GFP*

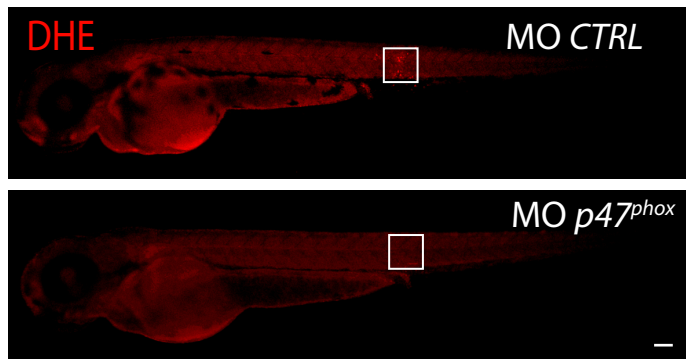

B

*Tg(mpx:GFP) - E. coli-GFP*

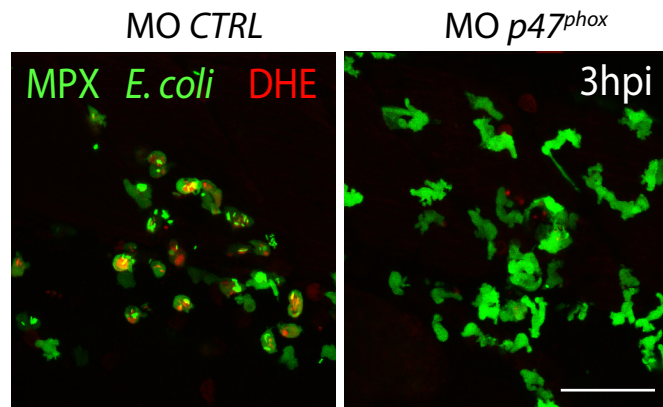

C

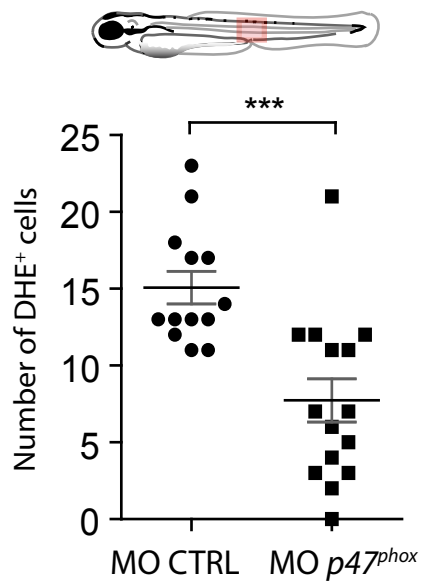

D

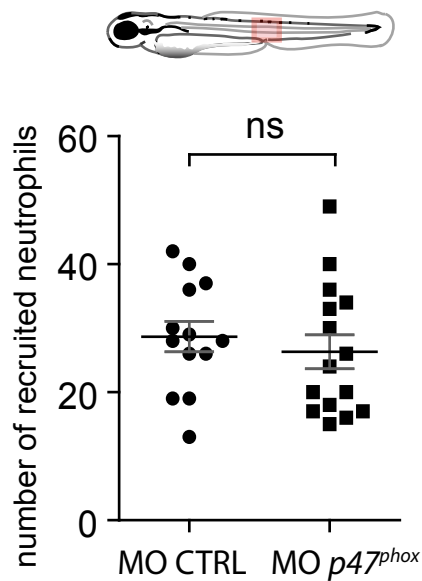

E

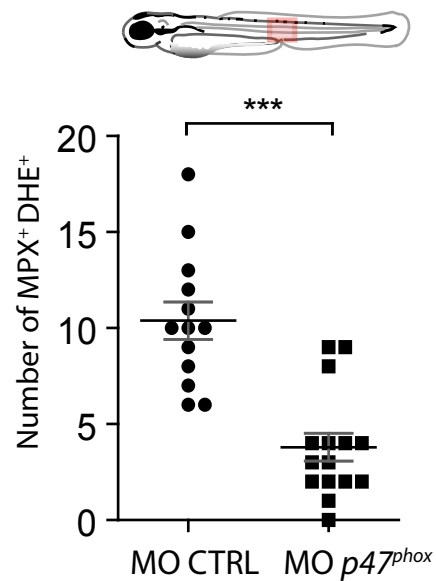

Supplement: S10 Fig — Tg(mpx:GFP) embryos were injected at the one cell stage with either p47phox morpholino (MO p47phox) or a control morpholino (MO CTRL). Morphants were infected in the muscle with GFP- E. coli and superoxide was detected with DHE at 3 hpi. (A) Larvae images are representative fluorescence images of DHE at 3 hpi. Scale bar: 100 μm. White boxes indicate the position of the confocal images in (B). (B) Representative overlay of GFP fluorescence (neutrophils+E. coli) with DHE fluorescence (maximal projections of confocal images) show superoxide in neutrophils in control morphants but not in p47phox morphants. Scale bars: 50 μm. (C-D) Dot plots are quantification of recruited DHE+ cells (C), recruited neutrophils (D), and recruited DHE+ MPX+ cells (E) in CTRL and p47phox morphants (mean number of cell/larva ± SEM, NMO CTRL = 13, NMO P47 = 13, Mann-Whitney test, two-tailed, ***p<0.001, ns: p>0.05 non significant). Diagrams show the counting region. (PDF) [file ppat.1007157.s010.pdf]
