# Supplementary material for: Neutrophils use superoxide to control bacterial infection at a distance
Source: PLoS Pathog. 2018 Jul 17;14(7):e1007157. doi: 10.1371/journal.ppat.1007157 (PMC6049935; doi:10.1371/journal.ppat.1007157)
Supplement: S3 Fig — Two dpf tg(mpx:GFP) embryos were infected in the notochord with low dose (< 3000 CFUs) (A, B), high dose (> 4000 CFUs) (C) or very high dose (>7000 CFUs) (D). (B) To decrease neutrophil density, tg(mpx:GFP) embryos were injected at the one cell stage with the csf3r morpholino and then infected in the notochord at 2 dpf with a low dose of red fluorescent E. coli-DsRed. (D) To increase neutrophil density, tg(mpx:GFP) embryos were injected at the one cell stage with the gcsfa over-expressing plasmid and then infected in the notochord at 2 dpf with a very high dose of red fluorescent E. coli-DsRed. Charts show the quantification of CFU (red-bar charts) and of the total neutrophil number (green-bar charts) at 0, 1 and 2 dpi (Mann-Whitney test, two-tailed, Nlarvae is indicated on the columns, *p<0.05, ** p<0.01 and ***p<0.001). Larvae images are representative overlays of fluorescence (green: neutrophils and red: E. coli) and transmitted light images at 2 dpi (asterisk: auto-fluorescence of the yolk). (PDF) [file ppat.1007157.s003.pdf]

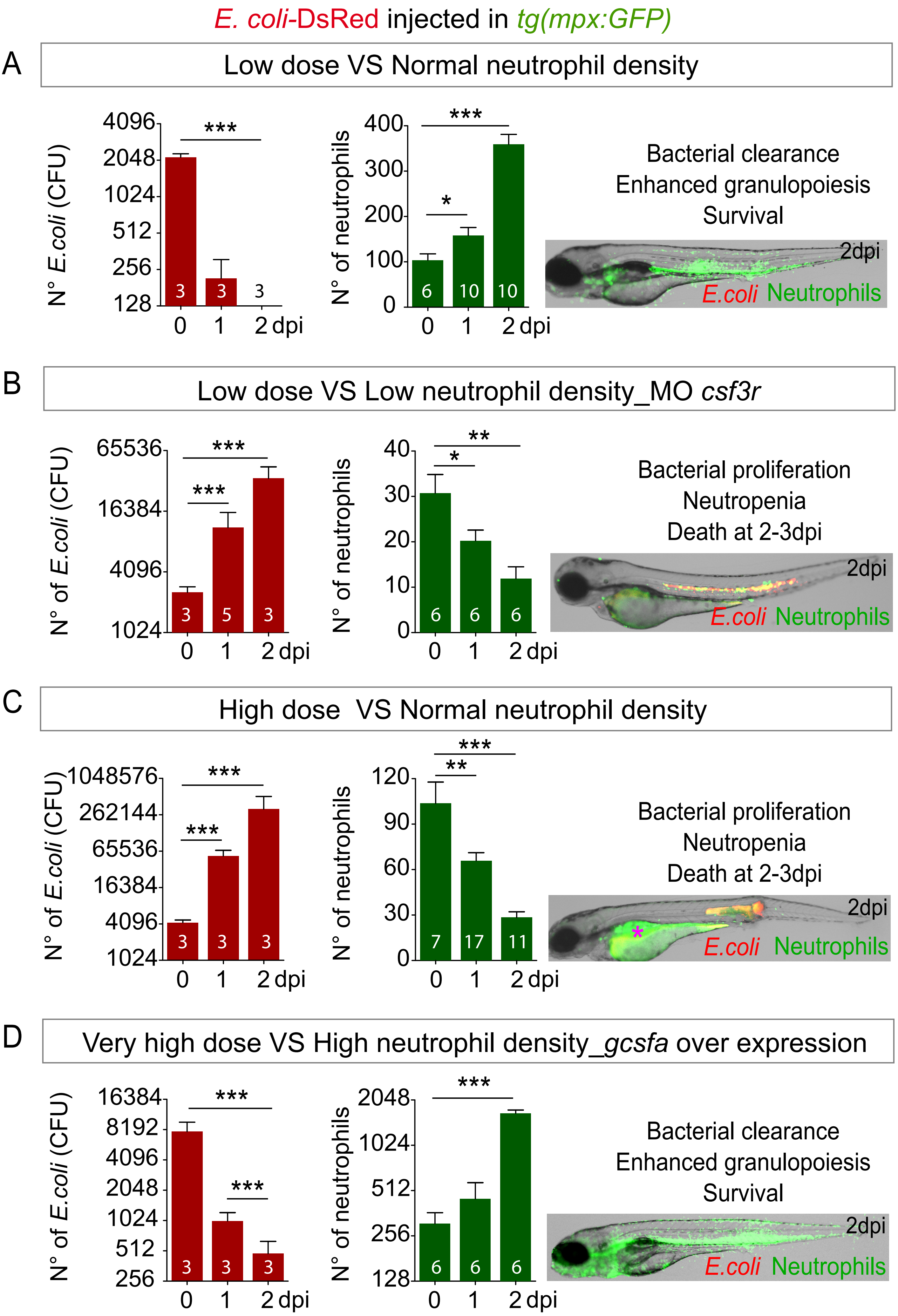
E. coli-DsRed injected in tg(mpx:GFP)
A
Low dose VS Normal neutrophil density
N° E.coli (CFU)
4096
2048
1024
512
256
128
***
3
3
3
0
1
2 dpi
N° of neutrophils
400
300
200
100
0
***
*
6
10
10
0
1
2 dpi
Bacterial clearance
Enhanced granulopoiesis
Survival
2dpi
E.coli Neutrophils
B
Low dose VS Low neutrophil density_MO csf3r
N° of E.coli (CFU)
65536
16384
4096
1024
***
***
3
5
3
0
1
2 dpi
N° of neutrophils
40
30
20
10
0
**
*
6
6
6
0
1
2 dpi
Bacterial proliferation
Neutropenia
Death at 2-3dpi
2dpi
E.coli Neutrophils
C
High dose VS Normal neutrophil density
N° of E.coli (CFU)
1048576
262144
65536
16384
4096
1024
***
***
3
3
3
0
1
2 dpi
N° of neutrophils
120
90
60
30
0
***
**
7
17
11
0
1
2 dpi
Bacterial proliferation
Neutropenia
Death at 2-3dpi
2dpi
*
E.coli Neutrophils
D
Very high dose VS High neutrophil density_gcsfa over expression
N° of E.coli (CFU)
16384
8192
4096
2048
1024
512
256
***
***
3
3
3
0
1
2 dpi
N° of neutrophils
2048
1024
512
256
128
***
6
6
6
0
1
2 dpi
Bacterial clearance
Enhanced granulopoiesis
Survival
2dpi
E.coli Neutrophils
